# Poly(*N*-isopropylacrylamide)-Based Hydrogels for Biomedical Applications: A Review of the State-of-the-Art

**DOI:** 10.3390/gels8070454

**Published:** 2022-07-20

**Authors:** Mohammad Javed Ansari, Rahul R. Rajendran, Sourav Mohanto, Unnati Agarwal, Kingshuk Panda, Kishore Dhotre, Ravi Manne, A. Deepak, Ameeduzzafar Zafar, Mohd Yasir, Sheersha Pramanik

**Affiliations:** 1 Department of Pharmaceutics, College of Pharmacy, Prince Sattam Bin Abdulaziz University, Al-Kharj 11942, Saudi Arabia; 2 Department of Mechanical Engineering and Mechanics, Lehigh University, 19 Memorial Drive West, Bethlehem, PA 18015, USA; rajendranrahulr@gmail.com; 3 Department of Pharmaceutics, Yenepoya Pharmacy College and Research Centre, Yenepoya (Deemed to be University), Mangalore 575018, Karnataka, India; mohanto111@gmail.com; 4 School of Bioengineering and Biosciences, Lovely Professional University, Jalandhar-Delhi, Grand Trunk Road, Phagwara 144001, Punjab, India; unnatiaggarwalsmile@gmail.com; 5 Department of Applied Microbiology, Vellore Institute of Technology, School of Bioscience and Technology, Vellore 632014, Tamilnadu, India; kingshukpanda7@gmail.com; 6 I.C.M.R.—National Institute of Virology, Pune 411021, Maharashtra, India; kishoredhotre27@gmail.com; 7 Chemtex Environmental Lab, Quality Control and Assurance Department, 3082 25th Street, Port Arthur, TX 77642, USA; ravimannemr@gmail.com; 8 Saveetha School of Engineering, Saveetha Institute of Medical and Technical Sciences, Chennai 600124, Tamil Nadu, India; deepakarun@saveetha.com; 9 Department of Pharmaceutics, College of Pharmacy, Jouf University, Sakaka 72341, Saudi Arabia; zzafarpharmacian@gmail.com or; 10 Department of Pharmacy, College of Health Science, Arsi University, Asella 396, Ethiopia; mohdyasir31@gmail.com; 11 Department of Biotechnology, Bhupat and Jyoti Mehta School of Biosciences, Indian Institute of Technology Madras, Chennai 600036, Tamil Nadu, India

**Keywords:** PNIPAM, hydrogels, drug delivery, tissue engineering, smart polymer, wound healing

## Abstract

A prominent research topic in contemporary advanced functional materials science is the production of smart materials based on polymers that may independently adjust their physical and/or chemical characteristics when subjected to external stimuli. Smart hydrogels based on poly(*N*-isopropylacrylamide) (PNIPAM) demonstrate distinct thermoresponsive features close to a lower critical solution temperature (LCST) that enhance their capability in various biomedical applications such as drug delivery, tissue engineering, and wound dressings. Nevertheless, they have intrinsic shortcomings such as poor mechanical properties, limited loading capacity of actives, and poor biodegradability. Formulation of PNIPAM with diverse functional constituents to develop hydrogel composites is an efficient scheme to overcome these defects, which can significantly help for practicable application. This review reports on the latest developments in functional PNIPAM-based smart hydrogels for various biomedical applications. The first section describes the properties of PNIPAM-based hydrogels, followed by potential applications in diverse fields. Ultimately, this review summarizes the challenges and opportunities in this emerging area of research and development concerning this fascinating polymer-based system deep-rooted in chemistry and material science.

## 1. Introduction

The importance of hydrogels as functional biomaterials in biomedical applications started in the early 19th century [1,2]. As per the report by Wichterle and Lim, hydrogel was initially used in biomedical applications as tissue scaffolds and contact lenses, but later, in the early 20th century, it was also used as high aqueous-swellable crosslinked polymeric networks [3]. Typically, hydrogels are fabricated via physically or chemically crosslinked hydrophilic polymeric macromolecules in a 3D network that can imbibe some water molecules and suspend their movements through physical and/or chemical linkages [4,5,6]. Hydrogels have become the center of attention in the application of chemical, biological, and pharmaceutical domains during the last two decades due to several intrinsic properties: biocompatibility [7], biodegradability [7], readily adjustable, hydrophilic characteristics [3], high absorption capacity [8], etc. Hydrogels substantially enhance the temporal and spatial delivery of macromolecular drugs, small molecules, and growth factors in several biomedical fields and have also had comprehensive clinical usage [9]. Hydrogel fabrication via natural/synthetic hydrophilic polymers drives the system to be more swellable and enhances the absorption capacity through attachment of hydrophilic functional groups, e.g., amide, amino, carboxyl, hydroxyl, etc.; to the polymeric chain. However, drug delivery using hydrogels has not been free of obstacles, but continuous advancements are helping to settle on a hydrogel design competently conformed to specific drug delivery [10,11]. Due to several limitations of using natural hydrophilic polymers in hydrogel preparation, e.g., low thermal stability [12], absorption capacity [13], gel strength [3], etc.; suitable synthetic polymers are being considered as a biomaterial for hydrogel preparation. Furthermore, several mechanical properties of polymeric hydrogels can be modulated via physicochemical reactions, altering the chains of the polymer with stimuli-responsive functional groups to enhance the physical and mechanical resistance, which are significant attributes to be considered when creating delivery systems [3,14]. In developing an efficient hydrogel system, these characteristics are positively interconnected; from biomaterial selection to developing a gel blueprint, it is typically required to assess the whole system.

Stimuli-responsive hydrogel systems, often known as “smart” or “intelligent” hydrogels, are a unique class of hydrogel due to their high swelling and gelation capacity [15]. Such hydrogels are competent in responding to environmental stimuli, e.g., pH, temperature, light, electrical field, and ionic concentration, by following a reversible change in the volume or a sol–gel phase transition [16,17,18,19]. Several thermoresponsive polymers develop gel at temperatures less than lower critical solution temperature (LCST). Positive thermosensitive hydrogels can shrink below and dissolve above the upper critical solution temperature (UCST). The solution formation process is reversible, and there is more swelling in the case of chemically crosslinked polymers when the temperature is lowered below the LCST. Negative thermosensitive hydrogels transform from sol to gel above the LCST, suggesting that they could be exploited to make hydrogels with delayed release, longer skin retention time, and minor systemic side effects [20]. Some previous extensive review articles also summarize the various theories and mechanisms of thermogelation [21,22,23]. Smart materials have demonstrated numerous promising applications in an aqueous medium. The nature of thermoresponsive hydrogels is similar to living tissue and hence could be utilized as an efficient carrier or delivery system in the biomedical field. This property efficiently simulates the sensitivity and responsiveness of biomolecules in in vivo microenvironments, which is a massive benefit in producing innovative biomedical composites for technical purposes. Thermo-sensitive polymeric materials can incorporate hydrophilic as well as hydrophobic structures of their parts, and the phenomenal temperature responsiveness is acquired from the complex interplay of the monomer’s hydrophilic and hydrophobic parts. Temperature alters the association of hydrophilic and hydrophobic components with water molecules, increasing solubility and causing the sol–gel phase transition of the crosslinked polymer [24].

This review concentrates on the specific properties, mechanism of phase transition in thermoresponsive PNIPAM, and the parameters impacting gel behavior. Further, a comprehensive review of recent investigations of PNIPAM-based hydrogels in diverse biomedical applications is presented. It focuses on numerous application domains, including tissue engineering, drug delivery, and wound dressing, plus broad fundamentals from material selection to greater efficiency hybridization techniques.

## 2. Brief on PNIPAM-Based Hydrogel

PNIPAM is a widely utilized negative thermosensitive polymer (as it has increased solubility with lowering of temperature, causing volume phase transition by forming hydrogen bonds) that has currently sparkled a lot of scientific inquisitiveness [25,26]. In the aqueous medium, PNIPAM assembles a stretched spiral elastic shape; polymer molecules form hydrogen bonds with each other due to hydrogen bonding with molecules of water. It has both hydrophobic isopropyl (–CH(CH3)_2_) side groups and hydrophilic amide (–CONH–) groups in the structure. PNIPAM has an LCST of 32 °C, which is somewhat lower than human body temperature of 37 °C, and it can be modified by copolymerizing with some distinct hydrophobic or hydrophilic polymers to develop thermo-sensitive, in situ hydrogels. [27,28,29]. The temperature-responsive behaviors are primarily due to connections between these groups and solvent molecules. As a result, PNIPAM converts from solution to gel state as the LCST reaches human body temperature [30,31,32,33,34]. Additional hydrophilic monomers increase the polymer’s hydrophilicity and lead to more significant interactions with water, resulting in a higher LCST, whereas copolymerization with more hydrophobic monomers reduces LCST [35]. This property makes PNIPAM suitable for biomedical applications, e.g., controlled wound dressings, tissue engineering scaffolds, and drug delivery systems [36,37]. Notwithstanding these appealing benefits, PNIPAM-based hydrogels nevertheless have certain drawbacks, including limited biodegradability [38], weak mechanical strength [39], insufficient drug loading capacity [40], and immediate release [39,41,42], etc.; limiting the feasibility in drug delivery.

Several drugs can be efficiently encapsulated into the hydrophilic polymers by using a lower temperature to dissolve the drug in the solvent to ensure efficient delivery, raising the temperature slightly above the LCST for hydrogel appearance. Furthermore, maintaining precise temperature control permits site-specific drug delivery and delayed release. The PNIPAM solution can be rapidly applied to the skin and activated by utilizing body temperature to create a 3D hydrogel that can help restore tissues by encouraging cell proliferation and differentiation while preserving the homeostatic activity of cells. The temperature-dependent modulation of gel coating and reversible sol-to-gel can be utilized to develop adherence to the skin and its dissociation for wound treatment. Other features of PNIPAM, in conjunction with thermosensitivity, include adjustable geometries and nontoxicity, both of which are advantageous for biological applications [43]. Wound dressings play a crucial part in lesion care, and their effectiveness impacts how quickly a wound heals [44]. That a warm, moist environment promotes faster healing has been widely demonstrated in several empirical investigations in both in vivo and in vitro wound healing [45,46]. Various medical studies also recommend that dressings should be translucent to allow easy observation, and that they have the ability to retain fluids [47,48,49]. Aside from the properties described above, zwitterionic hydrogels have lately gained popularity as nonadherent wound dressings due to their high resistance to cell attachment, protein adsorption, and microbial adherence [50,51]. For wound dressing, PNIPAM can be utilized to retain the skin adhesion and differentiation of gel coatings. Its thermosensitivity is complemented by LCST adjustability, low toxicity, and [48] variable structure. [52], making it suitable for biomedical applications [43].

Many contemporary tissue engineering techniques rely on the use of a material scaffold. These scaffolds act as an artificial extracellular matrix (ECM), allowing cells to be organized into a three-dimensional framework and stimulating the growth and creation of desirable tissue [53]. The scaffold material and qualities required vary greatly depending on the tissue of concern and the individual application [54]. Thermoresponsive polymers are extensively employed in tissue engineering in two ways: platforms for cell proliferation and injectable solutions for in situ scaffolding [55].

The primary disadvantage of PNIPAM-based hydrogels in drug delivery is the incorporation of hydrophobic drugs in the water-loving polymeric core [56], since mostly hydrophobic drugs are typically and effectively used in disease treatment. Along with this, the tensile strength [39] of these hydrogels is weak, which may sometimes lead to early drug release before arrival at the specific site [57]. Several researchers are working painstakingly to improve the mechanical characteristics of PNIPAM hydrogels to fix the reported shortcomings. Hybridization of PNIPAM-based composite hydrogels with appropriate materials such as inorganic/metal nanoparticles [58], organic self-assemblies [40], and several other polymeric material incorporations/composite formations are some reported viable techniques for achieving this goal [59,60]. A novel polymeric block could regulate the thermal sensitivity of the resulting hydrogel by adjusting the hydrophilic/hydrophobic equilibrium of PNIPAM. Alternatively, a novel polymeric component can affect the hydrogel network’s chemical rigidity and morphology, modulating the copolymeric hydrogel’s mechanical properties [61,62,63]. The successful composite formation of PNIPAM with various materials, e.g., hyaluronic acid (HA) [64,65], zinc oxide (ZnO) nanoparticles [66], silver nanoparticles [67], hydroxyapatite (HAp) nanoparticles [68], carbon nanoparticles [69], etc.; for tissue engineering and wound healing has been reported in several investigations [70,71,72]. Adequate biocompatibility, higher tensile strength, customizable temperature responsiveness, multi-drug loading capability, targeted drug delivery, and controlled drug delivery are among the unique and/or improved features of composite hydrogels [43]. The inclusion of some other polymer inside the hydrogel matrix to make interpenetrating polymeric network system (IPN) hydrogels for biomedical application [73] is another useful way to alter the functionalities of a hydrogel. The polymeric networks in IPN hydrogels can efficiently adjust mechanical characteristics, swelling/deswelling behavior, and drug loading/release pattern [74].

## 3. Unique Properties of PNIPAM

Physically crosslinked hydrogels can be assembled via ionic/hydrogen bond formation, Van der Waals forces, hydrophobic interactions, crystalline structure, etc. [75]. The strong hydrogen bonding between the polymeric chains in the hydrogel may promote drug release, which can be further controlled via the type of solvent, degree of sonication, solution temperature, polymer concentration, etc.; in hydrogel formulation. Another method, crosslinking, has been most commonly utilized to overcome biomaterial physicochemical and mechanical limitations via strong interconnections between the reaction molecules. Furthermore, ionic interactions can be initiated at room temperature and physiological pH to overcome constraints [76]. Bifunctional crosslinking agent-initiated hydrogels are generally developed via several techniques, e.g., condensation polymerization, irradiation using high-energy ionizing radiation such as electron beams, gamma rays, or X-rays, chain-growth polymerization, etc. [76].

In biomedical science, PNIPAM-based hydrogels have versatile applications for the effective therapeutic delivery of molecules by modulating substance movement in a medium, altering thermo-controlled dimensions [77]. A monomeric structure of PNIPAM commonly identified by isopropyl and amide moieties maintains LCST at ~32 °C in aqueous conditions. PNIPAM hydrogels are developed by crosslinking themselves or their derivates, exhibiting a reversible and extreme volume phase transition towards LCST through swelling/shrinking [78]. Along with the changes in size during the transition phase, there are also several other changes in many properties, such as hydrophilicity [79,80], transparency [81], and apparent electrostatic permittivity [82]. The mixing of polymer in a solvent leads to polymeric dissolution at a specific temperature (T), which the negative ΔG_mix_ of the Gibbs free energy equation can well indicate. The involvement of thermodynamics in the method forms hydrogen bonds that create significant negative enthalpy change of mixing (ΔH_mix_ < 0), contributing to dissolution. Lowering PNIPAM’s temperature below LCST develops a counterbalance between the hydrogen bonds of hydrophilic amide groups and molecules of water, leading to hydrophobic interactions among isopropyl groups and hence collapsing the PNIPAM chain to avoid water contact [78].

PNIPAM hydrogels exhibit a slow response to environmental temperature variations due to the formation of the thick skin surface, decreasing the outward diffusion of water molecules and causing hydrogel collapse at temperatures above LCST [83,84,85]. Likewise, the swelling rate of hydrogels is relatively slower at temperatures lower than LCST. Therefore, improving the rate of thermoresponsiveness of established PNIPAM hydrogels has become a research subject [86]. Some physical strategies have been involved in enhancing the thermal responsiveness of PNIPAM hydrogels, such as phase separation [87], porogen [88], freezing [83], vacuum-synthesis [89], and interpenetrating polymer networks [90]. Along with these, alternate chemical approaches have also been introduced, which include the addition of hydrophilic moieties [91], grafting freely mobile hydrophilic moieties [92], and reversible addition–fragmentation chain transfer (RAFT) polymerization [93] to attain a rapid response rate. Stimuli-responsive hydrogels require mechanical properties for various applications. Hence, to improve the PNIPAM hydrogel‘s mechanical properties, some techniques have been implemented, such as an interconnected polymer network [94], a hydrogel with two networks [95], a hydraulic ring for slides [96], nanoporous PNIPAM hydrogel [97], and copolymerized PNIPAM hydrogel [98]. Free radical redox polymerization of PNIPAM hydrogels is relatively weak and can even be affected using standard mechanical testing equipment [99].

Takigawa et al. determined PNIPAM hydrogel’s Young’s modulus (E0) for the first time in 1997. They found a stress–strain graph of the collapsed and swelled gel to be linear in a specific hardness strength test, where the E0 was a hundred times more for the collapsed state. The swelled and collapsed conditions were found to have fracture strains of 35% and 75%, respectively [99]. The collapsed state was found to have a higher E0 than the swollen state, as the collapsed gels had an increased number of crosslinks. The change in E0 at the initial stage of the scaffold was probably due to rapid relaxation shown by the macroporous hydrogel, which remains constant for a more extended period during cellular growth [100].

PNIPAM hydrogel has inadequate mechanical performance in the highly swelled state, prominently identified as a disadvantage in drug delivery. Its nonbiodegradable nature has led to surgical excision after the release of the drug. Removing the device by typical surgical operations is also challenging if it is too light, as it might break down during handling. However, PNIPAM hydrogel’s lack of controlled release capability, i.e.; leading to the release of impregnated drugs within 24 h, is a severe reason for the possibility of being restricted as a drug carrier [101,102,103]. A swelled PNIPAM hydrogel with a decreased polymeric mass per unit volume explains its poor mechanical characteristics and increased drug release rate. The decreased polymeric mass per unit volume results in the fast release of impregnated drugs because of the gel’s open pores and low mechanical characteristics. Rapid drug release from the drug reservoir is because of the weak intermolecular bonds of swelled PINPAM hydrogel [90].

In an aqueous phase at a certain temperature range of about 32 °C, for drug delivery, the PNIPAM hydrogel could be combined with bioactive components to form a solution. The introduction of polymers becomes possible in that particular physical condition. The subcutaneous injection of the polymer-loaded gel leads to sustained release and an immediate increase in physiological temperature (to about 36.5–37.5 °C). The biodegradability of the hydrogel leads to the release of encapsulated bioactive compounds, initially in the body via diffusion and later on by a mixture of diffusion and mechanical breakdown [104]. Both physical and chemical properties (melting point, temperature, glass transition storage modulus, crystallinity, etc.) are responsible for the biodegradability of the polymer [105]. The reduced biodegradability of PNIPAM hydrogel has restricted its use in clinical practice. Different crosslinking agents and/or biodegradable polymers or native polymers, including poly(amino acids) [106], polysaccharides [107], proteins [108], and synthetic polymers including poly(esters) [109], poly(caprolactone) [38], and poly(ethylene glycol) [110], have now been analyzed for the development of biodegradable PNIPAM hydrogels [104]. PNIPAM is also highly biocompatible with animal cells [104]. Cao et al. explored the use of PNIPAM–chitosan copolymers for ophthalmic drug delivery. The copolymer was utilized for encapsulation of timolol maleate molecules for around 12 h to effectively lower intraocular pressure (IOP). The in vivo study of PNIPAM–chitosan for thermo-sensitive hydrogels confirmed non-cytotoxicity, hence furnishing new insights into glaucoma therapy along with several other eye illnesses [111].

Biopolymers or artificially degradable compounds help alter the chemical composition of PNIPAM to obtain biodegradability and biocompatibility effectively. In a study by Das et al.; covalently crosslinked PNIPAM hydrogels utilizing NIPAM as the monomer, dextrin as the biopolymer, potassium persulfate (KPS) N,N′-methylene bisacrylamide (MBA) as the promoter, and N,N′-methylene bisacrylamide (MBA) as the crosslinker were effectively synthesized. Ciprofloxacin and ornidazole could be administered in a controlled manner by the novel PNIPAM hydrogel, as it is nontoxic and biodegradable [112].

## 4. Phase Transition for PNIPAMs

The polyacrylamide structure is mostly crafted with a hydrophilic amide group (almost 90%) and -C-C- hydrophobic portion. However, poly(*N*-isopropyl-acrylamide) and other N-substituted acrylamide polymers (PNIPAM) have balanced hydrophilic and hydrophobic regions below LCST. The gel-polymer/water system’s total energy is lowered due to hydrophobic polymers enveloped by water molecules below LCST [113]. The solvation and transition capacity of PNIPAM in cold water increases when the temperature is raised off its LCST (LCST ≈ 32–34 °C), leading to the “coil to globule” of the polymeric chain’s (CG) transition. The CG transition is in charge of phase inversion into rich layers. Polymeric/water phases further exhibit volume phase transition (VPT) [114]. A loss in entropy of water molecules enveloping the hydrophilic polymeric chain is counterbalanced by an increase in enthalpy owing to hydrogen bonding between the hydroxyl groups surrounding the polymeric chain’s hydrophobic sections. Hydrophobic hydration is a process that allows a hydrophobic polymer to stay hydrated in an aqueous environment. If the temperature is increased above LCST, water molecules leave the polymer chain and form a globule structure. As a result, the PNIPAM–polymer is hydrated, and a definite volume phase transition is observed. The phase separation initially occurs due to PNIPAM molecule incorporation into larger aggregates [115,116] via several mechanisms and factors, e.g., dewetting caused by solvent fluctuations, cooperative hydration [117,118,119], the aqueous medium’s energy state [120], endothermic heat [121], precipitation polymerization [122], etc. The hydrogen bond between water molecules and PNIPAM is weaker due to the temperature rising above LCST, leading to the formation of an unstable solution. Further, the transitions of the PNIPAM–polymer can be confirmed by FTIR spectroscopy; the hydrodynamic diameter of PNIPAM gel affects the volume phase transition, causing dehydration of the polymeric gel [114].

The dependency of LCST on molecular weight and concentration of PNIPAM polymer in H2O and D2O can be confirmed via dielectric relaxation spectroscopy with small- and wide-angle x-ray scattering (SWAXS) (DRS) to show phase transition. Several investigations have reported a strong effect of interpenetration between the diverse chains of PNIPAM at high concentration decreasing correlation length (ξ). The molecular weight of PNIPAM is entirely independent of ξ at higher concentrations due to PNIPAM chains’ crammed state [116]. Interface formation and interchange aggregation are reported to be higher as a result of lowering the molecular weight of the polymer [116]. Several investigations have studied the phase transition effect due to LCST and confirmed peculiar behavior prevails with hydration [118,123,124,125]. The phase transition of the PNIPAM network can also be modulated by precipitation polymerization at 37−45 °C (near the LCST). Several analytical studies, e.g., atomic force microscopy (AFM) with photon correlation spectroscopy (PCS), etc.; have reported narrow, dispersed, spherical microgel and hydrogel volume phase transition behaviors [126,127]. Moreover, the phase transition property of PNIPAM-based hydrogel systems is advantageous due to its high mechanical strength and encapsulation capability, which has gained more attention in biomedical and tissue engineering applications [128].

## 5. Preparation of PNIPAM-Based Hydrogel Systems

**Figure 1:**
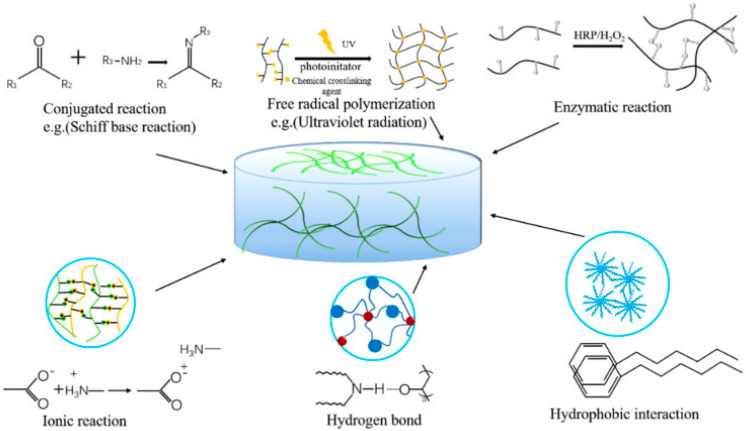
Physical and chemical crosslinking methods to prepare hydrogel systems for biomedical application [128].

The ideal hydrophilic polymer-based hydrogel possesses good biocompatibility and cell viability, excellent mechanical strength (especially stiffness for tissue engineering), high adhesion, moisture retention, promotion of cell proliferation, and high absorption capacity of the fluid for tissue engineering and wound healing [129]. Hydrogels are hydrophilic/hydrophobic monomeric unit-based systems that can be crosslinked via several techniques (e.g., physical and chemical crosslinking) to produce an elastic structure that can be affected/modulated via monomer, initiator, and crosslinker selection [130]. The synthesis or preparation of hydrogels with various functional properties can be initiated with physical and chemical crosslinking [129]. Several physical crosslinking methods (as displayed in Figure 1), e.g., ionic interaction, hydrophobic bonds, protein interaction, etc.; may improve the toughness and self-healing ability of the hydrogel system, which is further required for biomedical application. The hydrogels formed by ionic interaction, i.e.; dynamic interaction of the negatively charged groups or metal-ligand interactions, have improved self-healing, ionic conductivity, biological properties, etc. However, various limitations have also been reported, e.g., poor mechanical characteristics, complex/strong bonds between polymers, etc.; limiting the preparation technique’s usage [129]. Several investigations have been reported e.g., alginate/*N*-isopropylacrylamide (NIPAM) hydrogel [131], PNIPAM/poly(sodium acrylate) hydrogel [132], methacryloylchitosan/PNIPAM hydrogel [133], polyacrylamide/sodium alginate IPN hydrogel [134], etc.; based on ionic interaction of PNIPAM-based hydrogel systems for improving stiffness, cell viability, and mechanical integrity of the hydrogel system. Another physical crosslinking method involves the usage of dynamic hydrogen bond interaction, which is often unstable in an aqueous environment but is possible to rebuild after breaking; it can improve self-healing, cell biocompatibility, biodegradability, etc.; properties [135] via an IPN hydrogel preparation effective in wound-healing dressing. Several investigations, e.g., chitosan-poly (vinyl alcohol) (PVA) DN (double network) hydrogel [135], sodium alginate (SA)/polyacrylamide (PAM) semi-IPN hydrogel [136], hydrazide-functionalized PNIPAM/dialdehyde dextrin thermoresponsive hydrogel [137], etc.; have reported, based on dynamic hydrogen bond mechanistic, PNIPAM hydrogel preparations for biomedical application. Another commonly utilized physical crosslinking method, freeze–thaw, can form ice crystal and fabricate the polymeric chain around the crystals, followed by fabrication of a microporous structure while melting the crystals [138]. The freeze–thaw method can proliferate stem cells and ECM deposition [139], cell compatibility, and biodegradability [140] in the biomedical application based on the modulation of temperature, time, number of cycles, polymer contents, etc. [140,141]. Some recent investigations, e.g., PNIPAM hydrogel [142], PNIPAM/cellulose nanocrystal hybrid hydrogel [143], chitosan-graft-PNIPAM/PVA hydrogel [144], etc.; utilized the freeze–thaw physical crosslinking method and were reported to have high encapsulation efficiency, stimuli-responsive, adsorption capacity, etc. Hydrophilic polymers with hydrophobic end groups/side chains/monomers can be associated via physical crosslinking, resulting in high mechanical strength via strong hydrophobic interaction [129]. Several studies e.g., polyacrylamide (PAAm)/polyacrylic acid (PAAc)/PNIPAM hydrogel [145], poly(*N*-isopropylacrylamide) hydrogel [146,147], etc.; have been successfully prepared and reported high mechanical strength for biomedical application. The low mechanical stability and strength of physically crosslinked, reversible hydrogels may improve the utilization of chemical crosslinkers connected via covalent interactions. Diverse chemical crosslinking mechanisms have been reported, e.g., conjugation reaction, free radical polymerization, enzymatic reaction, etc.; where the hydrogel was formed via covalent bonds [148]. Conjugation occurred in mild conditions in the presence of Michael addition, Schiff’s base, Diels–Alder addition, etc.; which are green methods in the presence of condensate functional groups to improve biodegradability, transparency, and adhesiveness of the hydrogel system [129,148]. To overcome the limitations associated with the mechanical properties of hydrogel prepared by conjugation, free radical polymerization was reinforced via heating, ultraviolet radiation, energy radiation, electrolysis, etc.; to improve the swelling, porosity, and mechanical strength of the hydrogel system for biomedical application [148]. Several studies, e.g., PNIPAM/magnetite nanoparticles hydrogel [149], poly(*N*-isopropylacrylamide) hydrogel [150], PNIPAM–Ln(DPA)3 hydrogels [151], PNIPAM/gold nanocluster hydrogel [152], etc.; have utilized free radical polymerization for PNIPAM-based hydrogel preparation to improve mechanical strength and stability. The enzymatic reaction of natural polysaccharides in the presence of several enzymes, e.g., transglutaminase, tyrosinase, urease, horseradish peroxidase, etc.; occurs in very mild conditions and can retain the biological properties and improve the mechanical strength of the polymers utilized for hydrogel preparation. In various reports, e.g., *N*-isopropylacrylamide (NIPAM) and acrylic acid (AAc) hydrogel [153], vinylimidazole/PNIPAM) hydrogel [154], NaCMC/PNIPAM hydrogels [155], etc.; enzymatic crosslinking was seen to be effective for improving the mechanical stability and strength of PNIPAM-based hydrogels.

## 6. Formulation Approaches for Tailoring the Mechanical Behavior of PNIPAM Composite Hydrogel

The biomedical application of PNIPAM composite hydrogel must be considered based on biodegradability, stimuli-responsiveness, encapsulation efficiency, drug release behavior, mechanical strength, self-healing properties, etc.; which further depends on the selection of biomaterials, formulation strategy, and incorporation of other materials inside the hydrogel system, especially for drug delivery, tissue engineering, and wound healing. Several common crosslinking strategies have been utilized for hydrogel preparation to improve mechanical strength, but when it comes to the incorporation of drugs/molecules in the hydrogel system, the mechanical properties raise concern. Therefore, several techniques have been adopted to fabricate PNIPAM composite hydrogel, e.g., copolymerization with other monomers, fabrication/incorporation of polymeric nanoparticles, formation of IPN or nanocomposite hydrogel, etc.; where temperature, reaction conditions, chain functionality, drying method, degree of crosslinking, pH, and ionic strength can be tuned to improve the mechanical strength of the PNIPAM composite hydrogel [40,74]. Interpenetrating polymer networks (IPNs) are strong, noncovalently attached polymer networks consisting of more than two natural/synthetic polymers at a molecular scale and are difficult to separate without breaking chemical bonds. Semi-IPNs are the linear polymeric chain penetrating another crosslinked network, whereas in full IPNs, both the polymeric networks are crosslinked. The higher mechanical strength of IPN hydrogels is mostly via polymerization consisting of natural hydrophilic polymers alone or in a combination of synthetic polymers and natural proteins [40,74]. Double network (DN) hydrogels are a new category of IPN hydrogel with high mechanical strength (tensile strength: 1–10 MP, compressive fracture stress: 20–60 MPa, etc.) and higher moisture absorption capacity, effective for wound dressing [74]. IPN hydrogel may be crosslinked to such an extent that drug release will be retarded, and the degree of crosslinking also affects particle size, polydispersity, degree of polymerization, etc.; these are considered the main limitations [104,156] of the IPN hydrogel. Some recent investigations have reported that the PNIPAM IPN hydrogel improved various mechanical properties e.g., chitosan/PNIPAM IPN hydrogels [157], poly(acrylic acid)/PNIPAM IPN hydrogel [158], luteolin/hyaluronic acid (HA)/PNIPAM IPN hydrogel [29], etc. Another strategy to improve the mechanical properties of PNIPAM hydrogel is copolymerization by optical or chemical crosslinkers in the presence of an initiator in the solvent system. The copolymeric hydrogel had tuned biological and mechanical properties, especially the swelling ability of the polymeric networks, but was a poor bacterial barrier [159] and had weak tensile stress [160], unstable functional groups [161], noncovalent bonds [161], etc.; which may cause concern in biomedical application. A few recent investigations were reported on PNIPAM-based copolymeric hydrogel systems, e.g., Laponite^®^ platelets/PNIPAM hydrogel [162], polypyrrole/PNIPAM hydrogel [163], polyethylene glycol diacrylate (PEGDA)/chitosan (CS)/PNIPAM hydrogel [164], chitosan–*N*-2-hydroxypropyl trimethylammonium chloride (HACC)/PNIPAM hydrogel [165] etc.; where biological properties and mechanical properties were improved. Recently, nanoparticle-loaded composite hydrogels gained significant consideration due to high stimuli responsiveness, reversible deformation, one-pot synthesis method, notable biological and mechanical properties (i.e.; more elongation strength and higher compression); they were prepared via physical crosslinking or covalent integration. Several inorganic/metallic nanoparticles, e.g., ceramic, hydroxyapatite, carbon-based nanoparticles, etc.; can also be loaded into the PNIPAM-based hydrogel system due to their biological abilities and modulating mechanical strength, making them effective for tissue engineering and wound healing [74]. Despite several biological advancements of PNIPAM-based nanocomposite hydrogels, a few limitations have been reported, e.g., low biosorption in vivo, poor stimulation for the proliferation of new tissue, mechanical degradability in tissue engineering, etc.; which may limit utilization, especially in tissue engineering application [166]. Some recent studies, e.g., silver nanoparticles/(PNIPAM166-co-n-butyl acrylate9)-poly(ethylene glycol)-(PNIPAM166-co-n-butyl acrylate9) copolymeric hydrogel [167], cellulose nanocrystals/PNIPAM hydrogel [168], fibroblast growth factor/sodium alginate (SA)/PNIPAM nanogel [169], polydopamine nanoparticles/PNIPAM hydrogel, etc.; showed high stimuli responsiveness [167], encapsulation efficiency [168], storage modulus, and water absorption capacity [169] in tissue engineering and wound healing applications. Therefore, choosing the best material to form a hybrid system with PNIPAM requires extensive materials study, which can further modulate the properties and functionalities of PNIPAM-based hydrogels in biomedical applications.

## 7. PNIPAM-Based Hydrogels in Drug Delivery

Considering the thermoresponsive polymer poly (*N*-isopropylacrylamide) (PNIPAM) for its precise structure as well as properties, mainly because of temperature feedback that is close to human body temperature and that it can be fine-tuned to meet the imperative temperature–pH dual responsiveness of drug delivery systems [39], thermosensitive polymer free-radical polymerization was used to make PNIPAM. When exposed to organic solvents, the risk of drug denaturation and aggregation increases because hydrogels are made up of a substantial amount of water, and the crosslinked polymer network is decreased [170,171]. As a result of the polymer network containing crosslinks, hydrogels are solid-like and can possess various mechanical properties, for example, tunable stiffness, allowing their physical qualities to be harmonized with the human body’s different soft tissues; additionally, the stimuli-responsive hydrogels adjust their structural properties in response to peripheral stimuli and are also conditional based on environmental changes Stimulus-responsive hydrogels can alter phase or volume in response to stimuli and perform specified activities. A hydrogel that responds to stimuli such as near-infrared (NIR) may be modulated error-free by regulating the radiation intensity, light exposure time, and irradiation sites [172]. The crosslinked network can hinder the infiltration of several proteins; therefore, it protects against premature degradation of bioactive therapeutics by the inward diffusion of the enzymes. This is critically valuable for highly labile macromolecular therapeutics (meant for monoclonal antibodies and recombinant proteins), consisting of an escalating proportion of newly approved and under development drugs [171]. Interaction of PNIPAM with various other molecules comes up with many leads in therapeutic as well as biomedical studies. Strategies have been established to enhance the weak reaction to NIR irradiation of PNIPAM hydrogels, for instance by introducing NIR-responsive nano-components, including Fe_3_O_4_ nanoparticles, carbon nanotubes, graphene oxide nanosheets, and many more, into the hydrogel system.

Khan et al. utilized PNIPAM and carboxymethyl chitosan to create cytocompatible in situ crosslinked pH/thermo-dual sensitive injectable hydrogels (CMCS-g-NIPAM) via combining cold and free radical polymerization techniques. After subcutaneous injection in vivo, the produced formulations were intended to be utilized as drug depots for 5-fluorouracil (5-FU). Tube titling and optical transmittance studies were used to examine and validate the phase transition from the sol–gel state to the physiological range of temperature. The produced formulations had the most significant release in acidic pH at 25 °C, according to an in vitro release profile. An MTT assay was used to assess the toxicity of empty gel expressions on L929 cell lines, and the results verified cytocompatibility with no observable toxicity. In vitro cytotoxicity of drug-loaded hydrogel against HeLa and MCF-7 cancer cell lines demonstrated that 5-FU in depot state had controlled cytotoxicity compared to free 5-FU solution. In contrast to the loaded form, the IC50 values for free 5-FU (21.05 g/mL and 18.66 g/mL) were more significant. The results showed that the suggested porous in situ hydrogel formulations are pH/temperature-sensitive and capable of delivering regulated drug release systemically and intratumorally [173].

Kim et al. developed a double-crosslinked permeating polymer network (IPN) hydrogel with pH-sensitive hyaluronic acid (HA) and PNIPAM via radical polymerization and Michael addition, which aids in transdermal administration of luteolin while suppressing keratinocyte hyperproliferation in psoriasis. According to texture analysis and rheometry, the most adherent and stable crosslinked networks were found in an IPN hydrogel with a 3% crosslinking agent concentration. HA-PNIPAM IPN hydrogel successfully delivered luteolin to the epidermis and dermis, and monitoring the cytotoxic effects of the hydrogel for topical application revealed no toxicity. Therefore, IPN hydrogels might be used for luteolin transdermal administration for psoriasis skin alleviation [29].

Dynamic hydrogels are kept together by transiently stable connections, giving gels different features such as self-healing and shear-thinning [174]. They allow controlled regional and systemic delivery after treatment via a subcutaneous method for ongoing therapy in various disorders [173]. In this context, McInnes et al. investigated the long-term and temperature-dependent drug delivery of biodegradable porous silicon (pSi) loaded with fluorescent anticancer drug camptothecin (CPT) and coated with poly (*N*-isopropyl acrylamide-co-diethylene glycol divinyl ether) (PNIPAM-co-DEGDVE) or non-stimulus-responsive poly (amino styrene) (pAS) via long-term and temperature-dependent drug delivery by initiating chemical vapor deposition (iCVD). The pSi coated with non-stimulus-responsive poly (amino styrene) (pAS) also exhibited continual drug distribution profiles and independent of the release temperature. CPT was released in a burst form from the uncoated oxidized pSi control (21 nmol/(cm^2^ h)), and this was nearly comparable at temperatures between 25 °C to 37 °C, the LCST of the switchable polymer employed, PNIPAM-co-DEGDVE (28.5 °C). At 25 °C and 37 °C, the burst drug release of the pSi-PNIPAM-co-DEGDVE section was much more sluggish, at 6.12 and 9.19 nmol/(cm^2^ h), respectively. The total quantity of CPT distributed in 16 h was 10% greater at 37 °C compared to 25 °C for pSi coated with PNIPAM-co-DEGDVE (46.29% vs. 35.67%), showing that this polymer may be utilized to deliver drugs at high temperatures. The drug delivery patterns of pSi coated with pAS were likewise sustainable, although they were unaffected by the release temperature. These findings indicate that by functionalizing pSi using iCVD polymer films, it is possible to create long-lasting and temperature-responsive drug delivery. The iCVD method has several advantages, including applying the iCVD coating following drug loading to avoid triggering drug deterioration, which is frequently caused by being exposed to variables such as solvents or high temperatures. Importantly, because the iCVD approach is not affected by the surface chemistry or pore size of the nanoporous matrix being coated, it can be used on a wide variety of surfaces [175].

Surface introduced reversible addition-fragmentation chain-transfer (SI-RAFT) polymerization was used by Zheng et al. to produce a dual-responsive thermal- and pH-sensitive nanocarrier, where the core is silica and the shell is a block copolymer of poly (methacrylic acid) (PMAA) and PNIPAM. At extreme temperatures, the resultant SiO_2_-PMAA-b-PNIPAM elements are distributed independently in an aqueous solution, yet they are reversibly aggregated under acidic circumstances or at increased temperatures. These dual-responsive nanoparticles were used as carriers to transport the model drug doxorubicin (DOX) with excellent entrapment efficacy and loading capacity because of their small size (15 nm), lightweight cores, and high graft density (0.619 chains/nm^2^) generated using SI-RAFT polymerization. Both the temperature and pH of the surrounding media also influenced the rate of drug release. Furthermore, these nanoparticles preferentially precipitated under an acidic environment with rising temperatures, suggesting that they may be more capable of accumulating at tumor locations. Cytotoxicity investigation indicated that DOX-loaded nanoparticles were far more efficient against Hela cells, unlike free DOX at the same dosage. According to a cellular uptake experiment, SiO_2_-PMAA-b-PNIPAM nanoparticles may efficiently transport DOX molecules into the nucleus of Hela cells. These characteristics suggested that SiO_2_-PMAA-b-PNIPAM nanoparticles are a viable therapeutic option [176].

Additionally, Peralta et al. synthesized magnetic mesoporous silica nanoparticles with thermoresponsive polymer grafting for regulated drug delivery. For this purpose (PNIPAM-co-MPS), iron oxide nanoparticles with mesoporous silica shells were grafted with PNIPAM-co-3-(methacryloxypropyl)trimethoxysilane). Grafting and polymerization of as-prepared nanoparticles was achieved in a single step, followed by a radical polymerization technique. Using this approach, the polymer was effectively grafted onto the silica shell, leaving the mesopores empty for drug loading. To evaluate the efficacy of the fused nanoparticles as a thermosensitive drug delivery vehicle, ibuprofen was employed as a standard drug. In vitro drug delivery experiments were performed between 25 °C to 40 °C on the polymer’s LCST (PNIPAM-co-MPS). There was a significant contrast of 80% in ibuprofen release at these two temperatures, with the drug being released quickly and completely at 40 °C. These results suggest that the thermoresponsive copolymer acts as a barrier to the temperature-controlled release of the drug encapsulated within the mesopores. As a result, co-MPSs are thus promising magnetic and thermoresponsive nanocarriers for regulated medication delivery [177].

**Figure 2:**
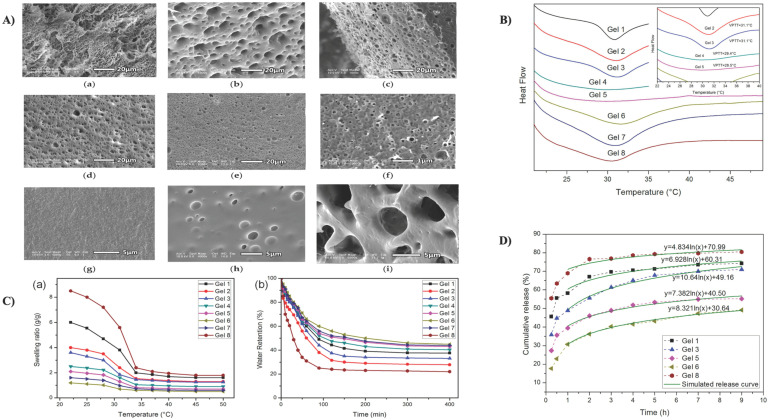
(**A**) SEM images of (a) Gel 1; (b) Gel 2; (c) Gel 3; (d) Gel 4; (e) Gel 5; (f,g) Gel 6; (h) Gel 7; and (i) Gel 8. (**B**) DSC analysis of gel phase transition characteristics. (**C**) (a) Temperature-dependent swelling ratios of gels between 20 and 50 degrees Celsius; (b) gel deswelling behavior at 60 degrees Celsius. (**D**) The simulated release curves and release behaviors of 5-FU from gels in PBS (pH 7.4) at 37 °C [177].

In another investigation, Li et al. widely studied a PNIPAM-MAPOSS hybrid hydrogel established on the presentation of acrylolsobutyl polyhedral oligomer silsesquioxane (MAPOSS) into the PNIPAM matrix in the presence of polyethylene glycol, which was prepared via radical polymerization (as shown in Figure 2A). To alter the pore size, PEG was employed as a pore-forming agent. MAPOSS lowered gel swelling ratios (as shown in Figure 2B) and reduced the LCST, triggering the hydrogels to shrink away at reduced temperatures (as shown in Figure 2C). However, its hydrophobicity aided in increasing the temperature reliability. The insertion of stiff MAPOSS into the polymer network raised the compressive modulus of the hydrogel by around tenfold [178]. The fused hydrogel can operate as a drug carrier for 5-fluorouracil (as shown in Figure 2D) and might have prospective applications in additional biomedical fields. 5-fluorouracil (5-FU) is an essential anti-cancer agent and perhaps the most extensively used in clinical anti-pyrimidine therapies [179]. It has an excellent therapeutic impact on gastrointestinal cancer and other malignant tumors. However, its fast metabolism in the body may diminish its therapeutic implications [180].

Accordingly, systems such as, PNIPAM@ magnetic NPs (Fe_3_O_4_)/5-fluorouracil (5FU) and oxaliplatin (OXA)- acrylic acid (AA), 3-butenoic acid (3BA) or allylamine (AL) (pNIPAM@ Fe_3_O_4_/5FU/OXA-AA, 3BA, AL) [181], doxorubicin(DOX)-gold nanorods (GNRs)-PNIPAM@poly (d, l-lactide)-poly (ethylene glycol) (PLA-PEG) (DAPP)micelles (DOX-GNRs-PNIPAM@PEG-PLA,DAPP) [182], vancomycin (VANCO)/SF-Na Alg NPs/PNIPAM@epidermal growth factor (EGF) (VANCO/SF-Na Alg NPs/PNIPAM@EGF) [183], PNIPAM- superparamagnetic iron oxide nanoparticles (SPION)- etoposide (SPION-PNIPAM-Etoposide) [184], NaALG-g-P(NIPAM_80_-*co*-NtBAM_20_)/Dulbecco’s modified Eagle’s medium (DMEM) (NaALG-g-P(NIPAM_80_-*co*-NtBAM_20_)/DMEM) [185] and many more have been designed by researchers in order to intensify the use of PNIPAM polymer in the field of drug delivery [186,187]. Table 1 depicts the latest investigations of PNIPAM-based hydrogels in drug delivery.

## 8. Gene Delivery

The introduction of transiently active nucleic acids for genetic engineering is a promising approach with prospective uses in the therapy of ailments ranging from tumors to communicable diseases to genetic syndromes. Reviving the expression of a defective protein, repairing faulty transcript splicing, and suppressing or regulating the expression of genes are all strong techniques that might have significant implications in biological discovery and treatment. In the last decade, substantial progress in enhancing gene delivery has been accomplished, and numerous products have entered the market [199]. While utilizing a polymeric transporter, the critical phases of gene delivery (correspondingly known as transfection) are (1) polymer with DNA complexation; (2) insertion of polymer/DNA composite (also known as polyplex) on top of cells for a duration of time frequently known as the transfection period; (3) elimination of composite from cells; and (4) incubation time. In most cases, complexation occurs at ambient temperature, while transfection and incubation occur at 37 °C (the cells’ body temperature to survive). Remarkably, thermoresponsive polymers are being utilized to improve transfection effectiveness by varying the temperature during the complexation, incubation, and/or transfection period [20].

Hydrogels constitute a class of biomaterials formed by self-assembling or crosslinking water-soluble polymers into a network [200]. Hydrogels’ porous and hydratable structure causes them to gel and swell in the biological milieu, allowing them to be injected locally without invasive surgery [201]. Hydrogels can be engineered via physical (such as ionic and hydrogen bonding and hydrophobic interactions) or chemical (such as Michael-type addition reaction or photo polymerization) crosslinking processes [202]. Additionally, they are designed to demonstrate modified properties to the tissue to be refurbished as 3D-bioprinted constructs [203,204] and/or injectable [205], stimuli-responsive [206], or adhesive systems [207,208,209]. Self-assembled PNIPAM-co-dAAc nanogels fused with GFP-green fluorescent protein remained extremely expressed in human mesenchymal stem cells (hMSCs) and are also a prospective material for gene delivery [178]. When a stimulus is applied to responsive gels, their properties change. The disruption or development of both chemical and physical crosslinks can cause this alteration, which may or may not be reversible [174]. Unique materials that respond to certain stimuli can be used to encapsulate drugs, proteins, and organisms on a macro and micro scale. Sensory factors such as pH and temperature modify these materials’ phases and hydrophobic interactions reversibly. These changes can lead to self-assembly of the materials, which enables controlled drug release and safe gene delivery into cells and tissues [210].

In a study by Zhang et al.; it was revealed that in comparison to traditional PNIPAM hydrogel, the swelling fractions of PNIPAM hydrogels with extremely permeable microstructures, organized utilizing hydrophobic polydimethylsiloxane (PDMS) along with sodium dodecyl sulfate, were better at room temperature. Their response rates were amplified intensely with the temperature above the LCST. For instance, an innovative hydrogel made in conjunction with a 40% PDMS template lost nearly 95% of its water in five minutes, but standard PNIPAM gel dropped just about 14% simultaneously. When liquid PDMS templates are added to reaction solutions, porous structures are created during polymerization/crosslinking, resulting in improved properties. Bovine serum albumin (BSA) and lysozyme as protein templates were physically absorbed into these micro-structured smart hydrogels. The experimental results revealed that—owing to the size exclusion effect, because BSA with a significant molecular weight has a lower loading efficacy than lysozyme—the loading effectiveness of both peptides in the porous hydrogel was significantly higher than that of the standard PNIPAM hydrogel. For instance, the load-up efficacy of BSA in the porous hydrogel is 0.114, which is around 200% more than the loading efficiency in traditional hydrogel (0.035). At 22 C, both BSA and lysozyme were totally liberated from the permeable hydrogel. Moreover, the rates of protein release from the porous hydrogel may be controlled by adjusting the ambient temperature. These newly developed porous materials exhibited excellent potential for protein or gene therapy since they present a mechanism to improve loading efficiency and manage the release patterns of macromolecular medications from hydrogels [211].

**Figure 3:**
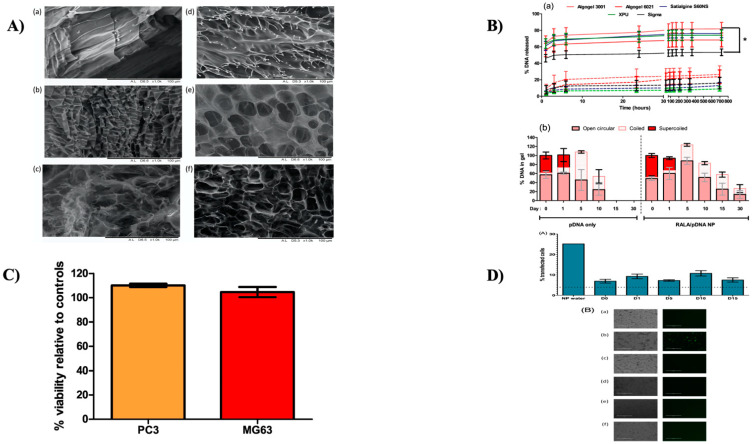
(**A**) SEM structural analysis of Alg-g-P (NIPAAm) hydrogels premised on (a) Algogel3001, (b) Algogel6021, (c) Satialgine S60NS, (d) SatialgineS900NS, (e) Sigma, and (f) XPU alginate. (**B**) As a pDNA and RALA/pDNA NPs delivery device, Alg-g-PNIPAM hydrogel was used. (a) For up to one month in DDW at 37 °C, hydrogels discharged pDNA (continuous line) at a faster rate (burst release) than RALA/pDNA NPs (dashed line). (b) DNA stability was sustained in algogel 3001-g-P (NIPAAm) hydrogels for uncomplexed pDNA for up to 10 days and complexed RALA NPs for up to 30 days; * *p*  < 0.05. (**C**) Degradation of algogel 3001-g-PNIPAM hydrogel at 70 °C in cell medium for 3 days caused cytotoxicity in PC3 and MG63 cells. (**D**) Evaluation of DNA transfection effectiveness after 15 days of incubation in Alg-g-PNIPAM hydrogel (A) flow cytometry analysis for transfection efficiency (B) The prevalence of green fluorescent protein-expressing cells for the various treatment groups. Reproduced with permission from [211], copyright Elsevier, 2017.

In another study, Chalanqui et al. developed Alg-g-PNIPAM hydrogel via free radical polymerization for the local release of nanoparticles of DNA as a possible medicinal mechanism for men diagnosed with castrate-resistant prostate cancer (CRPC). Considering that CRPC often spreads to the bone, initiating discomfort and death, combined with a slew of other skeletal-associated complications, a copolymer-based hydrogel was developed to extend curative DNA nanoparticles’ extended delivery. In milder circumstances, alginate grafted poly(*N*-isopropylacrylamide) hydrogels (Alg-g-PNIPAM)) develop 3D frameworks, making them ideal for the insertion of fragile biomolecules such as DNA. The effect of PNIPAM on copolymer qualities has been thoroughly explored, though the impact of alginate backbone features on copolymer properties has yet to be addressed. Six distinct Alg-g-PNIPAM hydrogels were created using 10% alginate, which differed in terms of molecular weight (MW) and mannuronate/guluronate (M/G) monomer ratio, and 90% NIPAM to create an injectable highly thermoresponsive hydrogel preparation for localized gene delivery (as shown in Figure 3A). Hydrogels with high MW or low M/G ratio alginate backbone tend to have higher stiffness than hydrogels with low MW alginate and a high M/G ratio. A complexed and meshed hydrogel network was also generated by hydrogels with a high M/G ratio, whereas hydrogels with a low M/G ratio developed a simpler architecture with the juxtaposition of Alg-g-PNIPAM sheets. These nanoparticles were made using plasmid DNA (pDNA) functionalized in conjunction with RALA, an amphipathic cell-penetrating peptide intended to penetrate cells efficiently. The rigidity, morphology, injectability, and deterioration of the Alg-g-PNIPAM hydrogel were all influenced by the alginate MW and M/G ratio. Algogel 3001, filled with RALA/pDNA NPs, exhibited the best properties for long-term use. According to the release profiles, RALA preserved the pDNA from degradation over 30 days (as shown in Figure 3C) and provided a persistent and regulated release from hydrogels when matched to pDNA alone (as shown in Figure 3B). After incubating it for 15 days in Alg-g-PNIPAM hydrogel, the efficacy of DNA transfection was evaluated. Flow cytometry analysis of the NPs incubated inside algogel 3001-g-PNIPAM hydrogel demonstrated constant transfection effectiveness of 10%, which was independent of the time of incubation (as shown in Figure 3D) [212].

In another study, Cao et al. utilized conventional radical graft copolymerization to integrate the thermosensitive polymer PNIPAM in the side chain of low-molecular-weight PEI to form an amphiphilic graft copolymer at 80 °C reaction temperature and encapsulated the TRP53 gene, a tumor suppressor gene involved in cell cycle regulation and programmed cell death [213]; pH-sensitive nanogels are ideal for delivering integrated payloads to specific target locations with altered environmental conditions caused by disease processes [214].

In another investigation, Fliervoet et al. conducted a study for offsetting electrostatic and hydrophobic interactions in thermosensitive plasmid DNA (pDNA)-loaded polyplexes for nucleic acid delivery. By using a hetero-functional PEG macroinitiator, NPD triblock copolymers composed of a thermosensitive poly(*N*-isopropylacrylamide) (PNIPAM, N), along with a cationic poly(2-(dimethylamino)ethyl methacrylate) (PDMAEMA, D) and a hydrophilic poly (ethylene glycol) (PEG, P) block with variable block dimensions were formulated. According to dynamic light scattering, the NPD polymers self-assembled into polyplex nanostructures with hydrodynamic diameters ranging from 150 to 205 nm at room temperature in HBS buffer. Polyplexes with a low-down N/P charge ratio of 1 accumulated when heated to 37 °C, whereas greater N/P charge ratios did not. Throughout all N/P ratios and extreme temperatures, stable polyplexes were produced when the cationic D block was comparatively lengthy compared to the thermosensitive N block. The overall viability of these polyplexes at 37 °C was further supported by H-NMR studies, static light scattering, and possible dimensions. Furthermore, the addition of thermosensitive blocks to NPD-based polyplexes increased cytocompatibility in HeLa cells when associated with PD-based polyplexes with comparable cargo delivery competencies [215].

Correspondingly, HOOC-PNIPAM -*b*-poly(2-(dimethylamino) acrylate)-C_12_H_25_/DNA (HOOC-PNIPAM-*b*-QPDMAEA-C_12_H_25_/DNA) [191], chitosan-*g*-PNIPAM) (CPN)@graphene oxide (GO)-cetuximab (CET)/irinotecan (CPT-11)@short hairpin RNA (shRNA) hydrogel (CPN@GO-CET/CPT11@shRNA) [216] and siRNA@poly(2-dimethylaminoethyl methacrylate) (PDMAEMA)/PNIPAM-PEG-PNIPAM hydrogel (siRNA@PDMAEMA/PNIPAM-PEG-PNIPAM) [217] systems were formulated by researchers and demonstrated promising results in gene delivery. Table 2 demonstrates the latest studies on PNIPAM-based hydrogels in gene delivery.

## 9. PNIPAMs Hydrogel in Tissue Engineering

### 9.1. Cartilage Tissue Engineering

Cartilage is a smooth, elastic tissue membrane that covers the heads of the bones in articulating joints and allows smooth joint motion. It is composed of chondrocytes that are involved in producing collagen fibers and proteoglycans (PG) [221]. Due to its avascular structure, cartilages possess a low rate of healing capacity [222]. Cartilage damage leads to arthritis, the most common cause of disabilities caused by cartilage-related disease in recent years [223]. A scaffold can be a biological or a synthetic microstructure that can provide a niche to accelerate cell growth and trans-differentiation into various phenotypes. Currently, scientists are interested in exploring new and more biocompatible scaffolds for tissue engineering and delivery systems. Thermosensitive hydrogels are the most preferred hydrogels in tissue engineering, and PNIPAM is the one that has been extensively studied [224].

Irregular degenerative cartilage defects are also accompanied by osteoarthritis. Osteoarthritis is a significant disease characterized by pain, cartilage degradation, and inflammation. The uneven composition of injured cartilage further complicates the engineering of appropriate scaffolds for fabrication. Hydrogels are extensively employed for three-dimensional (3D) stem cell culture, but hardly any are utilized for stimulating in situ cell aggregation for specific applications. In this context, Zhang et al. reported the synthesis of a neo-cartilage patch by examining the mechanism of mesenchymal stem cell aggregation within the p(NIPAM-AA) and forcing aggregates onto electrospun film [225]. These cell aggregates promoted chondrogenesis by increasing endogenous growth factor production and extracellular matrix (ECM) deposition inside the p(NIPAM-AA) hydrogel. These in situ-generated cell aggregates were bioengineered into a neo-cartilage patch that might be used to repair superficial irregular cartilage lesions.

Many attempts have been made to develop hydrogel-based synthetic cartilage, but unfortunately, none have achieved cartilage-like properties. In this context, Means et al. concocted a double network (DN) PNIPAM hydrogel that possessed superior mechanical properties similar to cartilage, with a thermal transition temperature adjusted beyond the physiological scale. They prepared the DN PNIPAM hydrogel by using poly (2-acrylamido-2-methylpropanesulfonic acid) (PAMPS) as a primary network and poly(*N*-isopropylacrylamide-co-acrylamide) [P(NIPAM-co-AM)] as another network [226]. PAMPS/P (NIPAM-co-AM)-DN hydrogels exhibited an outstanding cartilage-like characteristic, making them a good choice for cartilage replacement as synthetic cartilage grafts.

**Figure 4:**
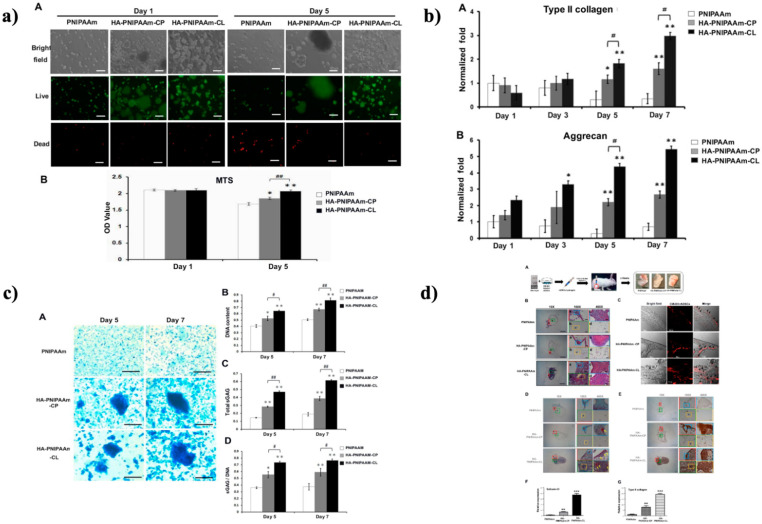
(**a**) The cytocompatibility and cell survival of rADSCs were improved by HA-modified PNIPAM hydrogels. Using (A) live and dead staining and (B) an MTS assay, cell survival of rADSCs encapsulated in PNIPAM, HA-PNIPAM-CP, and HA-PNIPAM-CL hydrogels was determined during days 1 and 5. (**b**) In rADSCs cultivated in PNIPAM, HA-PNIPAM-CP, and HA-PNIPAM-CL hydrogels for 1, 3, 5, and 7 days, the chondrogenic indicators of gene expression of (A) type II collagen and (B) aggrecan were detected. Collagen type II and aggrecan mRNA expression levels in rADSCs cultivated in HA-modified hydrogels are expressed and normalized in comparison to rADSCs cultured in PNIPAM hydrogels, which is designated as 1. (**c**) At days 5 and 7, there was enhanced cell aggregation and cartilaginous matrix sGAG production in rADSC cultured HA-modified PNIPAM hydrogels in vitro. (A) Glycosaminoglycans stained with Alcian blue (sGAG). (B) The DMMB assay was used to quantify the production of sGAG. (**d**) In vivo evaluation of the increase of neocartilage development in rADSCs/HA- PNIPAM-CL constructions using a rabbit model. (A) Illustration of the intraarticular injection of the rADSC/hydrogel constructions into the synovial cavity of rabbit knees. After 3 weeks, injected rADSC/hydrogel constructions were collected from rabbit synovial cavities and analyzed using (B) H&E staining, (C) confocal microscopy for pictures of bright fields, and CM-DiI-labeled rADSCs (red, arrows), (D) safranin-O fast green staining showing sGAG deposition (arrows), and (E) IHC staining for type II collagen synthesis (brown). Normalized relative to the PNIPAM group, which is defined as 1. Quantification examination of safranin-O staining (F) and type II collagen staining (G). Scale bar: 100 μm. (*), (**), and (***) represented *p*  <  0.05, *p*  <  0.01, and *p* < 0.005 respectively, in contrast with the PNIPAM group. (^#^), and (^##^) represented *p* < 0.05, and *p*  <  0.01, respectively in contrast with the HA-PNIPAM-CP group [226].

For tissue engineering applications, injectable thermoresponsive hydrogels enable excellent cell transport and low invasion. The ability of recently designed thermoresponsive poly(*N*-isopropylacrylamide) (HA-PNIPAM-CL) hydrogels modified with hyaluronic acid (HA) to enrich rabbit ADSC (rADSC) chondrogenesis in the synovial cavity of rabbits as well as in vitro was examined in a study by Wang et al. Physical interaction and chemical crosslinking were used to make HA-crosslinked PNIPAM (HA-PNIPAM-CL) and HA-mixed PNIPAM (HA-PNIPAM-CP) (as shown in Figure 4a). In vitro studies demonstrated that the HA-modified hydrogels equally dramatically boosted cell survival, chondrogenic marker gene expression (aggrecan and type II collagen), and sulfide glycosaminoglycan (sGAG) production in implanted rADSCs as compared to original PNIPAM. HA-PNIPAM-CL, on the other hand, had the best rADSC survivability and chondrogenesis (as shown in Figure 4b,c). The chondrogenic activities of HA-modified hydrogels on rADSCs were validated in vivo by injecting hydrogel-embedded rADSC assemblies into rabbit synovial cavities for three weeks and mapping using CM-DiI labeling. Type II collagen and GAG histomorphological staining were used to detect neocartilage development in the hydrogels. In the synovial cavity of rabbits, rADSC/HA-PNIPAM-CL induced greater hyaline cartilage development than rADSC/HA-PNIPAM-CP and rADSC/PNIPAM constructs (as shown in Figure 4d). These findings implied that the HA-PNIPAM-CL milieu was suited for enhancing ADSC chondrogenesis in articular cartilage tissue regeneration [227].

Recent advances in photocrosslinkable hydrogel synthesis have enabled the fabrication of native articular cartilage. However, various aspects, including selecting appropriate cell sources and biocompatible scaffolds for hosting cell support and proliferation, remain to be addressed. Chitosan is widely used as a bio-macromolecular adhesive [228]. It not only possesses a similar molecular structure to hyaluronic acid and glycosaminoglycans (GAG) but also mimics the natural microenvironment to improve chondrogenesis and modulates cell proliferation and differentiation [229]. In this regard, Mellati et al. described the potential role of chitosan-graft-poly(*N*-isopropylacrylamide) (CS-g-PNIPAM) hydrogel and micropatterning of 3D cell-laden as a technique for manipulating the cell shape and multi-zonal cartilage tissue engineering [230]. The hydrogel was capable of mimicking the superficial zone of cartilage and tended to be a suitable candidate for chondrogenic differentiation of mesenchymal stem cells (MSCs).

Electrospun hydrogels were used to make fibrous scaffolds. However, they tend to collapse when cells are seeded into them. An electrospun hybrid scaffold composed by integrating PEG-PNIPAM in conjunction with a biodegradable, eco-friendly polymer such as poly (ε-caprolactone) (PCL) might overcome this limitation. This hybrid scaffold had improved mechanical properties and enhanced the chondrogenic demarcation of human mesenchymal stem cells (hMSCs) [231]. In another analysis, Saghebasl et al. demonstrated a biodegradable thermosensitive hydrogel scaffold (PNIPAM-PCL-PEG-PCL-PNIPAM)/gelatin fabricated by TIPS (thermally induced phase separation). The in vitro study found that the scaffold provided a better survival rate for encapsulated cells and expressed cartilage-related genes. Hence, it serves as a potential approach to encourage the development of cartilage tissue [232].

Apart from this, other formulations that researchers have synthesized are Au NPs/chitosan/k-carrageenan/poly(NIPAm) [233] and vancomycin-loaded cellulose nanocrystals/Fe_3_O_4_/PNIPAm [168], which showed promising results in cartilage tissue engineering.

### 9.2. Bone Tissue Engineering

Bone is a hard tissue comprised of different bone cells containing osteoblasts and osteocytes [234]. The structural integrity of bone is threatened by physical injury. Gold standard medication for bone defects is an autologous bone graft. Nevertheless, the scope of autologous bone grafting is restricted by irregular shape, donor number, and immunogenicity. Therefore, bone tissue engineering (BTE) becomes crucial to overcome these limitations. Hydrogels are applied for bone regeneration, and various studies reported using modified hydrogels to increase new bone formation [235].

Due to the avascular nature of bone, the vitality of cells after implantation is a critical concern in bone tissue engineering. Trans-differentiation of human bone marrow-derived mesenchymal stem cells (hBMSCs) to an endothelial cell phenotype is achievable. This can be accomplished by combining the delayed distribution of vascular endothelial growth factor (VEGF) with osteogenic stimuli to increase both angiogenic and osteogenic differentiation [236]. Osteoclasts are involved in the secretion of bone morphogenetic proteins (BMPs) into the extracellular matrix during bone development. BMP-2, a member of the transforming growth factor (TGF) class, controls the development of chondrocytes and osteoblasts [234]. Following this, Muller et al. suggested the use of poly (NIPAM-co-DMEAEMA)/cellulose sulfate complex hydrogel for switchable release of bone morphogenetic protein (BMP-2) on demand for local bone healing and regeneration [237]. The authors reported that with the introduction of a thermoinducible delivery system, the release of BMP-2 from PNIPAM-DMAEMA/CS increases with increasing temperature. Overall results demonstrated that the delivery system has the potential to treat acute bone defects such as osteoporosis.

Hydroxyapatite (Hap) is an inorganic component of bone; synthetic HAP, Ca_10_(PO_4_)_6_(OH)_2_, a calcium phosphate, shows structural along with chemical similarities with the mineral phase of human bone and teeth [238]. HAP has been extensively used with hydrogel scaffolds for bone regeneration studies [239]. Thorpe et al. developed a laponite (crosslinked PNIPAM-co-DMAc) hydrogel delivery arrangement stacked with hydroxyapatite nanoparticles (HAPna) [240]. The study focused on the efficacy and in vivo safety of the hydrogel in rats. This resulting delivery system was biocompatible while promoting enhanced bone formation and assisting cell movement to stimulate incorporation with encircling bone. The in vivo and in vitro studies exhibited the induction of osteogenic differentiation without the need for supplementary growth elements in mesenchymal stem cells (MSCs).

Postmenopausal osteoporosis (PO) is caused by estrogen deficiency with age in females, which is distinguished by minimal bone mineral density (BMD) loss, causing bone fragility. Estrogen affects bone by increasing calcitonin production, which in turn inhibits bone resorption and lowers the sensitivity of bone mass to parathyroid hormone (PTH), sequentially reducing bone resorption. Menopause leads to estrogen deficiency, which impairs the normal bone turnover cycle. Therefore, the quantity of bone reabsorbed surpasses the total of bone deposited, ultimately leading to the net loss of bone [241]. Mesoporous hydroxyapatite (MHA) possesses meticulous pore size and optimized pore volume as well as exhibiting excellent biocompatibility [242]. Simvastatin (SIM), commonly employed in the treatment of hyperlipidemia, has recently gained popularity as a bone development medication. However, the hydrophobicity of SIM makes it challenging to release SIM sustainably and locally in vivo. Wu et al. fabricated PNIPAM brush-modified MHA nanoparticles loaded with simvastatin [243]. This system delivered a constant release of hydrophobic SIM and exhibited great potential for promoting osteogenesis.

In contrast, graphene derivatives have been shown to support stem cell attachment and differentiation [244]. Graphene oxide as a scaffold shows excellent biocompatibility, with its exceptional physicochemical properties, comprising brilliant hydrophilicity with hydrophilic functional groups. To exploit this property, Amiryaghoubi et al. invented a temperature-sensitive injectable hydrogel employing poly (*N*-isopropylacrylamide)-based copolymer/graphene oxide (GO) through chitosan (CS) as a natural polymer. The study found that prepared hydrogel was highly biocompatible for hDPSCs by providing a biomimetic ECM microenvironment for hDPSC proliferation, and thus could be used as a potential bioactive material for transplantation of hDPSCs. Developing an injectable bone-forming hydrogel would have broad clinical and economic benefits. Calcium phosphate cements (CPCs) are frequently used for bone regeneration applications. CPCs possess excellent biocompatibility with bone tissue. Unfortunately, CPCs are highly brittle and their usage is limited in load-bearing skeletal sites. To improve their toughness, Petre et al. suggested the incorporation of fibers as the enforcing component for CPCs by covalently modifying the surface of poly (vinyl alcohol) with PNIPAM brushes [245].

Likewise, many such formulations have been designed by researchers, which displayed promising results, such as (PNIPAM)/hydroxyapatite (HAp) scaffold [246], carboxymethyl xylan (CMX)-PNIPAm (CMX-PNIPAm) [247], etc.

### 9.3. Cardiac Tissue Engineering

In the current scenario, tissue engineering becomes a ray of hope for cardiac regeneration. Several studies have reported cardiac regeneration, such as providing mechanical support, cardio-protective molecule delivery, and others [248]. Hydrogels are an essential therapeutic approach in cardiac tissue engineering for various reasons. Concerning the cardiac environment, the properties of injectable hydrogels can be altered to provide physical, chemical, and electrical compatibility. To provide structural support to continuously contracting cardiac muscles, hydrogels with varying stiffness can be synthesized to enable their biocompatibility. Hydrogels may serve as a controlled delivery system for injecting genes directly into damaged cardiac tissue for tissue regeneration [248].

Myocardial infarction (MI), typically referred to as a heart attack, is a condition in which the heart muscle is permanently damaged. Fibrin-rich thrombus development, which inhibits blood flow in coronary arteries, is a significant cause of MI. When oxygenated blood flow returns to the tissue after a period of ischemia, ischemia reperfusion injury (IRI) occurs (hypoxia). This restoration causes inflammation and oxidative damage, which contributes to cardiac fibrosis, and lack of energy for cardiomyocytes leads to death. Cardiac function can be improved after an MI by injecting biomaterials into the infarcted heart’s ventricular wall and by reducing stress on the left ventricular wall of cardiomyocytes by mechanical load shielding.

**Figure 5:**
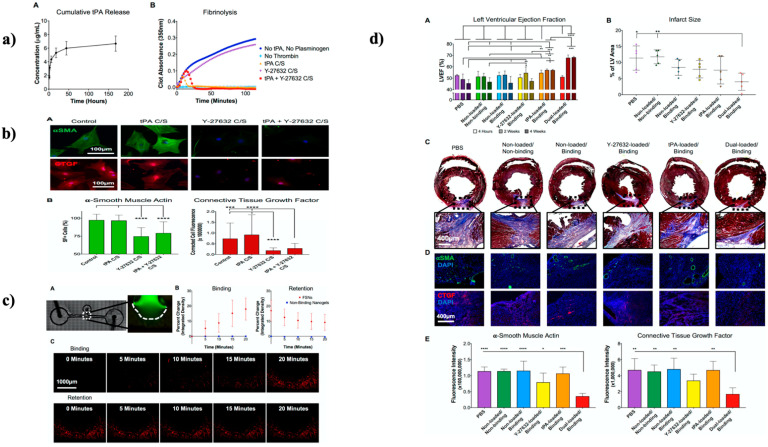
(**a**) Effective burst release of tPA is indicated by cumulative tPA release from C/S nanogels (A) and examination of fibrinolysis in the existence of drug-loaded nanogels (B). (**b**) The expression of fibrotic markers such as -SMA and CTGF on neonatal rat cardiac fibroblasts is reduced by introducing drug-loaded C/S particles in vitro (A). Percentage of stress fiber-positive cells for -SMA and adjusted total cell fluorescence for CTGF were used to quantify the results (B). (**c**) In vitro, FSNs adhere to and are maintained at fibrin clot borders at 1 sec-1 wall shear rates. A fibrin clot (green) was polymerized along the channel using PDMS molds (A). Particle binding (red) throughout 20 min and retention (C) during a 20 min buffer wash demonstrate deposition at fibrin clot sites, as measured by fluorescence intensity at the clot boundary (B). (**d**) In vivo dual-loaded FSNs augment left ventricular ejection fraction 2 and 4 weeks after I/R (A). Dual-loaded FSNs dramatically reduce infarct size (B) 4 weeks after damage, as measured by Masson’s trichrome staining and measuring blue collagen stain as a percent of the left ventricular area (C). Four weeks after I/R (D), dual-loaded FSNs significantly reduce -SMA (top, green) and CTGF (bottom, red) expression in vivo, as measured by immunofluorescence intensity (E). (*), (**), (***), and (****) represented *p* < 0.05, *p* < 0.01, *p* < 0.001, and *p* < 0.0001, respectively. Reproduced with permission from [248], copyright ACS, 2018.

In an investigation, Mihalko et al. used fibrin-specific poly (*N*-isopropylacrylamide) nanogel in a projected dual-delivery technique capable of reestablishing blood flow and simultaneously inhibiting cardiac fibrosis. This system consisted of tissue plasminogen activator (tPA) combined with a cell contractibility inhibitor (Y-27632); tPA, is an enzyme responsible for clot breakdown, and Y-27632 is essential in rho-associated protein kinase (ROCK) signaling pathway inhibition. Strategically, the dual delivery system design allowed for a preliminary release of tPA to soften the prime MI fibrin deposition, trailed by an additional constant release of Y-27632 (as shown in Figure 5a) to avert cardiac scarring through obstructive critical cellular replies intricated into the onset plus development of fibrosis. The in vitro studies showed fibrin degradation with decreased cardiac cell fiber formation (as shown in Figure 5b,c). In contrast, in vivo studies demonstrated improved ventricular ejection fraction and reduced infarct size (as shown in Figure 5d), advocating the value of this therapy for improving cardiac function after a heart attack [249].

Another technique that scientists explore is therapeutic angiogenesis. Angiogenic factors are used as therapeutic proteins to treat myocardial ischemia and increase perfusion to the surviving cardiomyocytes. Angiogenesis is triggered when angiogenic molecules such as VEGF (vascular endothelial growth factor) attach to the endothelial cell receptor, causing new blood vessels to develop. Due to rapid diffusion from the target site, poor constancy, and short half-lives of angiogenic factors, several dosages are required, which has the disadvantage of unrestrained vascular development at undesirable spots. In this context, Lee et al. coupled poly (serinol hexamethylene urea) (PSHU) with PNIPAM, and sulfonated groups were used to create an injectable sulfonated reversible thermal gel [250]. The electrostatic interaction amongst heparin sulfate coupled with angiogenic agents is exploited in this hydrogel. Heparin sulfate’s electrostatic interaction with angiogenic factors provides long-term transport, drug binding, receptor stability, and proteolysis protection while maintaining the bioactivity of the angiogenic factors. This blend was established for intramyocardial injection in conjunction with angiogenic factors such as VEGF to safeguard cardiac function following myocardial infarction. In vivo study exhibited promising results with the localized release of VEGF, improved vascularization, and potential role of anti-apoptotic impacts of the thermal gel.

It is complicated to select the optimal techniques for preventing myocardial necrosis and optimizing cardiac healing after a heart attack. Revascularization of the injured myocardium was the focus of the investigation. To avoid the MI–heart failure vicious cycle, it is also critical to limit overreactive and protracted inflammatory responses following cardiac injury, in addition to increasing vascularization (HF). In this regard, Rocker et al. proposed the use of multiple angiogenic factors (PDGF (platelet-derived growth factor) and VEGF) and anti-inflammatory cytokine (IL-10) to promote angiogenesis and restrict the detrimental impacts of MI [251]. Sulfonated reverse thermal gel (SRTG), a polymeric distribution system, is created from poly (serinol hexamethylene urea) (PSHU) coupled with PNIPAM with sulfonate groups. The system could sequentially deliver VEGF to initiate the angiogenesis process, followed by IL-10, which could suppress proinflammatory mediators to protect the heart from unnecessary inflammatory damage, and the final delivery of PDGF to stabilize newly formed vesicles and reduce their regression.

Further, following MI, injured tissue does not mend spontaneously, as mature cardiomyocytes have limited capacity to proliferate, and scar tissue is formed instead. The scar tissue that has replaced it is unable to conduct electrical or mechanical impulses, reducing pumping capacity even more. Vasoconstriction, hypertrophy, or enlargement of the cardiac muscles for increased pumping force and left ventricle enlargement help to stabilize reduced output. This extra complication may weaken the heart and lead to worsening cardiac function, eventually leading to heart collapse. Patients with end-stage heart failure have only two options for treatment: heart transplantation or mechanical ventricular assist devices (VADs). Lately, cardiac tissue engineering (CTE) has been widely investigated. Cui et al. evaluated the biocompatibility of PNIPAM-based-microgels to provide an optimized microenvironment for cardiac stromal cells (CSCs) with different surface charges and degrees of hydrophilicity and functional groups [252]. The study reported that a negative charge and more hydrophilic microenvironment of PNIPAM exhibited higher viability and proliferation of human cardiac stromal cells (hCSCs) and neonatal rat cardiomyocytes (NRCMs). Nanofibrous gelling microspheres (NF-GMS) are a copolymer of poly (l-lactic acid)-b-poly (ethylene glycol)-b-poly (*N*-iso-propylacrylamide). NF-GMS mimicked the extracellular matrix (ECM) and showed promising results for transplantation of human embryonic stem cell (hESC)-derived cardiomyocytes (CMs) for reducing infarct size in rats, leading to the substantial recovery of cardiac function [253].

Heart tissue possesses a complex three-dimensional (3D) structure. The myocardium chamber possesses a helical fiber with curved and hollow lumen organization. With few available fabrication techniques, it becomes difficult to recreate such complex carved structures in vitro. To address this need, Williams et al. established a fabrication technique for patterning the 3D structure of heart tissue by layering numerous layers of cells onto adaptable scaffoldings combined with casting them into cavity tubular hydrogels [253]. The complex 3D tubular hydrogels were constructed by layering individual sheets of cardiomyocytes on flexible thermoresponsive nanofabricated substrates (fTNFS), layer-by-layer stacking, and casting them into 3D tubular geometries with twisted molds. The resulting pre-patterned cell sheets after tissue casting promoted cellular configuration in 3D tissues. The results suggest that a mixture of internal as well as external signals may be essential to extend mature tissues. The flexible TNFS approach can be used for fabricating more advanced engineered tissues. In another investigation, PNIPAM- gelatin-based injectable hydrogel proved a prominent candidate for cardiac tissue engineering [254].

### 9.4. Lymphoid Tissue Engineering

The lymphatic system is made up of lymphoid tissue, which is majorly associated with fluid homeostasis and internal fluid drainage, lipid absorption, and immune cell surveillance [255]. Several complications related to the lymphatic system include lymphedema, cancer progression and metastasis, cardiovascular disease, impaired wound healing, and obesity. Tissue engineering has become an essential tool for providing both ex vivo research models and a therapeutic for alleviating lymphatic deficiencies [256]. Several studies have been done on engineering lymphatic capillaries, lymph nodes, and other nonspecific lymphoid organoids [257]. Hydrogels are majorly associated with lymphoid tissue engineering during the 3D cell culture matrices for modeling the lymphoid tissue, expanding hematopoietic–lymphoid cells, and incorporating growth factors during vascular network formation in in vivo and in vitro conditions. Thermo-responsive inverted colloidal crystal (ICC) hydrogel scaffolds are used for mimicking lymphoid cells as they consist of fully interlinked, tunable pore arrays, which provide both a large area and multicellular communications [258]. PNIPAM is used to create several thermoresponsive hydrogels for designing ICC hydrogel scaffolds which will undergo a volumetric change over a physiological temperature range. Thermoresponsive cell culture dish helps control cell adhesion in various temperature ranges [259]. PNIPAM-based hydrogels help deter mechanical and chemical disintegration of the hydrogel scaffold, allowing the pore-entrapped hematopoietic-lymphoid cell to be released without breaking the scaffold [258]. Recently, Kwak et al. designed a thermoresponsive ICC hydrogel scaffold by polymerization between PNIPAM hydrogel scaffolds and a nanogel crosslinker as a potent strategy for releasing pore-entrapped hematopoietic-lymphoid cells without breaking the scaffold [258]. According to the findings, ICC PNIPAM-NG allowed the recapitulation of bone marrow microenvironment function, which created an environment for hematopoietic cell proliferation and retrieval via temperature changes.

### 9.5. Intestinal Tissue Engineering

The gastrointestinal tract is associated with various functions such as ingestion, digestion, nutritional element absorption, and waste excretion [257]. Tissue engineering for intestinal tissues has become an emerging field as it is majorly associated with replacing functional tissue containing biological activity and biodegradability. Intestinal stem cells also possess great potential for tissue engineering and are widely grown for biological research and several applications. Lately, Dosh et al. investigated alternative hydrogels that could support Caco-2 plus HT29-MTX cells and the development of a small intestine villi 3D model under in vitro conditions [260]. They found that l-PNIPAM hydrogel scaffolds maintained the 3D culture of two human colon adenocarcinoma cells, stimulated the cells to form villus-like structures, and helped with differentiation into native small intestinal epithelium. This research indicates the potentiality of PNIPAM hydrogel to deliver a 3D culture and could be used to investigate several diseases. A different approach was used by the authors in another experiment, where they used PNIPAM hydrogel to design a 3D coculture model of Caco-2 and HT29-MTX cells to regulate its possibility in exploring inflammatory bowel disease [261]. The study demonstrated that PNIPAM hydrogel helped maintain the 3D model even after treating the cell with IL-1β, TNF-α, and hypoxia for 1 week [261]. Later, in another study set, Dosh et al. showed the potential of i-PNIPAM hydrogels in providing support for the formation of enteroids in vitro as a scaffold, and of cell differentiation of mice isolated small intestinal crypts along with Lgr5^+^ stem cells [262]. Previously, different materials had been used, such as PLA, Matrigel, PGA, and collagen type 1 as a cellular scaffold, but those showed several limitations in various areas. This study showed that synthetic i-PNIPAM has many advantages, such as supporting crypt cell seeding and adhesion, cellular survival and differentiation, and others [262]. Table 3 demonstrates the latest studies on PNIPAM-based hydrogels in various tissue engineering.

## 10. PNIPAM Hydrogels for Wound Dressings

Wound dressings are a conventional clinical treatment to keep the wound moist and warm to help with healing. Although there is no ideal wound dressing material, cotton wools, gauzes, and adhesive bandages are generally used. These materials merely offer general safeguard of wounds but do not help in a speedy recovery [40]. A significant problem associated with these materials is that they can dry out and harden, which can cause secondary injury upon removal [40]. Currently, many wound-healing materials have been developed, but hydrogels have been identified as an ideal wound dressing component. PNIPAM hydrogels have some additional advantages for wound dressing, as these hydrogels are thermosensitive. Maintaining the moisture level in the wound area with maximum mechanical strength is an essential criterion for a wound dressing component. Due to its transparency, PNIPAM hydrogel allows supervision of the healing process [40]. It also helps keeping a moist environment in the wound area.

**Figure 6:**
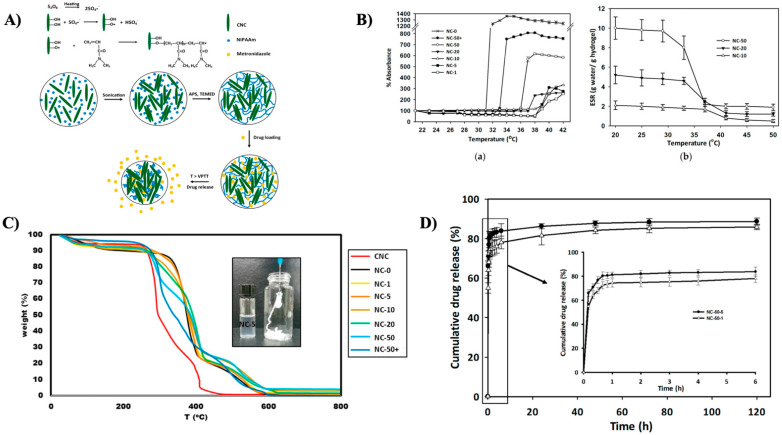
(**A**) The synthesis of thermoresponsive poly(*N*-isopropylacrylamide) (PNIPAM)-cellulose nanocrystal (CNC) hybrid hydrogels and a graphic depiction of the structural framework of drug release and drug load. (**B**) Thermoresponsive characteristics of PNIPAM-CNC hybrid hydrogels: (a) volume phase transition temperature (VPTT) profiles of hydrogels NC-0, NC-50+, NC-50, NC-20, NC-10, NC-5, and NC-1, with temperatures of 32, 34, 36.2, 37.5, 38.5, 39, and 39 degrees Celsius, correspondingly; (b) equilibrium swelling ratio (ESR) of PNIPAM-CNC hybrid hydrogels at various temperatures. (**C**) TGA thermograms of a pure CNC sample and produced PNIPAM-CNC hybrid hydrogels with varied levels of CNC content revealing thermal degradation characteristics. (**D**) Metronidazole (MZ) release profile from NC-50 hydrogels in phosphate-buffered saline (PBS) with pH 7.4 at 37 °C in vitro [142].

Recently, Zubik et al. synthesized thermoresponsive hydrogels comprised of poly(*N*-isopropylacrylamide) (PNIPAM) using free-radical polymerization and strengthened both noncovalent and covalent connections with cellulose nanocrystals (CNC) devoid of any added crosslinkers (as shown in Figure 6A). The quantity of CNC introduced determined the characteristics of PNIPAM-CNC hybrid hydrogels. The hydrogels’ thermal stability deteriorated as the CNC concentration increased. The viscous and elastic moduli of hydrogels rose with increasing levels of CNC, indicating greater mechanical capabilities of the hydrogels according to rheological measurements. Furthermore, hydrogel incorporation validated the idea that CNC strengthened the hydrogels, as enhanced CNC content improved the material properties after injection. The PNIPAM-CNC hybrid hydrogels had a volume phase transition temperature (VPTT) of 36° to 39° Celsius, equivalent to human body temperature (as shown in Figure 6B,C). To investigate drug loading and release for wound dressing applications, researchers used metronidazole, an antiprotozoal and antibiotic frequently employed in topical infections. At room temperature, the hydrogels had a significant drug-loading efficiency and a rushed drug delivery, accompanied by a steady and continuous release at 37 °C (as demonstrated in Figure 6D). These findings indicated that recently discovered therapeutics incorporated in injectable hydrogels could be helpful to wound dressing materials [143]. Hathway et al. also designed a PNIPAM-co-allylamine grafted material on nonwoven fabric that had increased mechanical strength and became more prominent for use as a bandage [271].

In another investigation, Li et al. tried to prepare alginate Ca^2+^/PNIPAM interpenetrating hydrogels onto a cotton fabric surface using three different grafting methods [272]. They found that fabric-supported hydrogels have the ability to maintain wound area breathability and comfort. They also supported the moist environment and controlled drug release [272].

Previously, some studies reported the bacteriostatic property of AgNPs. Although proper care should be taken for using AgNPs, high exposure of AgNPs to cells can be toxic and cause serious damage. Recently, AgNPs have been incorporated into PNIPAM hydrogels to make a wound dressing with antimicrobial activity. This strategy also reduces the direct exposure of AgNPs into the cell, resulting in less cytotoxicity. In that context, Qasimet et al. encapsulated AgNPs within PNIPAM and pNIPAM-NH2 polymeric nanoparticles via the reduction of silver nitrate. The result exhibited substantial bacteriostatic activity against both Gram positive and Gram negative bacteria on a quantitative basis of both nanoparticle size and silver nitrate used [273].

In another study, Gao et al. synthesized an Ag@PNIPAM nanocomposite that demonstrated highly effective antibacterial activity against *Staphylococcus aureus* and a high recovery rate against burn wounds [274]. Antimicrobial resistance is a primary focus of biological science in this situation. Recently, Liu et al. designed a first aid wound dressing component by combining sodium polyacrylate with triangular AgNPs@PNIPAM, which could be an effective strategy for microbial resistance. The dressings were created in an intelligent way in that they can regulate temperature, antibacterial properties, and the release of nanoparticles [275]. The race for developing methods of speedy wound recovery is still ongoing or under investigation. Lately, Blacklow et al. developed a PNIPAM-based hybrid hydrogel that could heal the wound in a natural process [276]. They prepared an active adhesive dressing (AAD) using PNIPAM, alginate, carbodiimide, silver nanoparticles (AgNPs), and chitosan. The components showed several advantages such as high stretchability, toughness, strong tissue adhesion, high antimicrobial activity, and speedy skin wound healing [276].

## 11. PNIPAM for Bioelectronics

Many bioelectronic technologies require seamless integration onto the surfaces of vital organs, where the interfaces provide soft mechanical coupling and efficient electrical/optical/thermal and chemical exchange. PNIPAM has been widely used to develop next-generation bioelectronic interfaces in recent years.

Electronic devices and computers can be used to create therapeutic body–machine interfaces using electrically active tissues such as the brain, heart, and skeletal muscle. In 2012, Tiwari et al. developed a PNIPAM-CNT-polyaniline-based three-dimensional nonwoven scaffold to check the effect on cell growth and viability [277]. They reported that the microfabric scaffold provides an excellent surface for cell growth and proliferation. This finding opened a new path in the field of tissue regeneration. Recently, Bagherifard et al. designed a PNIPAM-based drug delivery system that allowed drug release and growth factors to treat skin disorders [278]. They developed the platform using thermoresponsive microparticles, a flexible hydrogel layer, a heater for microparticle stimulation, a controller, and a power source. PNIPAM was used in this study due to its thermoresponsive behavior, increasing the possibility of modulating drug release rate through a temperature-dependent manner. A dermal patch that utilizes thermoresponsive drug microcarriers encapsulated within a hydrogel layer was attached to a flexible heater with integrated electronic heater control circuitry. The engineered patch covered the wound and allowed drug delivery by electronically adjusting the temperature of the hydrogel layer. In another study, Chen C. et al. developed a hydrogel patch for diabetic wound treatment [279]. Due to its thermoresponsive nature, they used PNIPAM-based hydrogel loaded with vascular endothelial cell growth factor as a filler, which could be released in response to temperature stimulus. Few studies report the association of PNIPAM with nucleic acid, as it consists of hydrophilic amido and hydrophobic isopropyl groups. A study by Lee A.W. et al. generated PNIPAM-DNA supramolecular complexes, transforming the PNIPAM into a semiconductive matter [280]. In another investigation, PNIPAM was used to generate PIPAAM-nucleobase supramolecular complexes (PSCs) by hybridizing with five different nucleobase units (adenine, thymine, uracil, guanine, and cytosine) [281]. Similarly, neat PNIPAM was transformed into a semiconductor from an insulator after hybridizing with nucleobases. Such improvements in the conductivity of hydrogels could lead PNIPAM’s use as a novel material in bioelectronics.

Recent development in bioelectronics has provided tremendous opportunities to allow diverse methods for monitoring and treating long-term disease. However, current electronics–tissue biosystems are characterized by weak physical interactions, rigidity, and dislocation during long-term application. Electrically conductive hydrogels exhibit flexibility (arising from hydrogel) and electrical conductivity (arising from conductive components) [282]. Further, electrically conductive hydrogels are divided into polymer-based (PPY, PANI, and PT) [283,284,285,286,287], metal-based (silver nanowires and gold nanoparticles) c [286,288,289,290], ionic (lithium chloride and sulfonate) [291,292,293], and carbon materials based (carbon nanotubes and graphene) [290,294,295,296]. Polypyrrole (PPy), being a conducting polymer, is extensively studied as an electrochemical actuator. It demonstrates outstanding potential in a variety of applications, including electrochemical sensors [297], mechanical actuators [298], supercapacitors [299,300,301], and artificial muscles [302,303,304]. However, despite the wide applications, its brittle and rigid nature may not suit its application in biological systems. The use of pNIPAM hydrogel provides more than x2 actuation and exhibits better optimization, bending, and out-of-plane deformations. In another study, Xue et al. demonstrated the use of hydrogel-based bioelectronics to enable implantable bioelectronics for monitoring and treating long-term diseases such as hypertension, diabetes, and Parkinson’s. Moreover, in vivo reports indicate promising results by exhibiting applicability. This hydrogel-based bioelectronic interface enabled recording of physiological parameters (blood pressure and electromyograph) while also showing trigger-detachable properties without causing skin, tissue, or organ damage [304].

## 12. Conclusions and Future Perspectives

According to the literature review, PNIPAM is the most explored and preferred thermoresponsive polymer, with applications ranging from tissue engineering to medication delivery and wound dressings. The significant advantage of PNIPAM is that it has an LCST of about 32° and is easily triggered by body temperature to give a phase transition. This unique property makes PNIPAM-based hydrogels highly suitable for various applications. PNIPAM hydrogels still have a few drawbacks, including low mechanical strength, limited drug loading capacity, low biodegradability, etc. Their slow response speed and low mechanical strength have hindered further development. Recently, scientists have tried designing PNIPAM-based hydrogels for tissue repair and regeneration. However, they remain unsuccessful because of their nonbiodegradable nature, which poses the problem of long-term bioaccumulation. The development of PNIPAM-based cell sheeting has also encountered complications. The chain length and film thickness of PNIPAM and its copolymers vary depending on the type of cells. As a result, further development is restricted to achieving a stable condition suitable for many cell types. Researchers have devised several effective strategies to overcome these challenges, including improving biocompatibility, mechanical strength, and biodegradability, initiating multi-stimulus interactions, or enabling multidrug encapsulation and advanced drug loading amounts. Polymerization methods such as live radical polymerization, ATRP, EB-irradiation-induced polymerization, and RAFT or light-induced polymerization have been used to create a range of thermoresponsive PNIPAM-modified surfaces. Another strategy focused on producing copolymeric hydrogels by linking PNIPAM with other polymer blocks with unique properties. Additionally, random or block copolymerization of ionic or hydrophobic monomers with PNIPAM provides additional functionality to the thermoresponsive interfaces. Such thermoresponsive surfaces provide a microenvironment for fabricating cell sheets, followed by modulating cell adhesion and detachment. The layer-by-layer technique has recently emerged as a potential surface modification technique. To deliver medications, a porous hydrogel structure can act as a matrix for drug loading while also protecting drugs from the environment. Furthermore, the porosity of the gel matrix can be adjusted by altering crosslinking density. Copolymerization is an essential approach in the context of the delivery system. A new drug delivery system can be developed by creating a surface with different adhesion kinetics. However, the most state-of-the-art PNIPAM-based hydrogels are still at the developmental stage and far from clinical practice.

The invention of a successful, intelligent hydrogel delivery system requires excellent material engineering and, more than ever, an interdisciplinary approach involving polymer scientists, medical practitioners, chemists, and pharmacists.

## Figures and Tables

**Table 1:** Current studies on PNIPAM-based hydrogel in drug delivery.

Hydrogel Composition	Drug	Preparation Technique	Key Features	References
PNIPAM/poly (ε-caprolactone) 8 dimethacrylate (PCLDMA)/bisacryloylcystamine (BACy)	Levofloxacin	Polymerization	The thermosensitive and biodegradable hydrogels were made from PCLDMA as a hydrolytically degradable unit along with a hydrophobic unit, with BACy as reducible degradation combined with a hydrophilic unit. The advantages of both thermoresponsive and biodegradable polymer systems was amalgamated.	[38]
PNIPAM/PPCN	Chemokine SDF -1 alpha	Sequential polycondensation and radical polymerization	The thermosensitive and biodegradable hydrogels with inherent antioxidant properties for the effective distribution of therapeutics was observed.	[110]
PNIPAM/Hyaluronic acid (HA)	Melatonin	Copolymerization	PNIPAM with HA increased the interrelatedness in microscopic structure with mechanical and chemical properties, and the hydrogels were highly adaptable to liquid/gel conversion temperatures which aid the improved support of the microenvironment for cell expansions and aggregates.	[188]
PNIPAM/Hydroxypropyl guar-graft-poly(N-vinyl caprolactam)	Ciprofloxacin	Graft polymerization	In situ covalent crosslinking of HPG-g-PNVCL copolymer with nano-hydroxyapatite (n-HA) by using divinyl sulfone (DVS) as a crosslinking agent to achieve HPG-g-PNVCL/n-HA/DVS composite material.	[189]
PNIPAM/poly(methacrylic acid)	Doxorubicin	Distillation precipitation or emulsion precipitation copolymerization	The PMAA/PNIPAM-1 microgel, prepared with the moderately-swollen PMAA cores for thicker PNIPAM shells via distillation precipitation copolymerization in acetonitrile, displayed more efficient pH and temperature-independent dual-stimuli responsive controlled releasing performance, while the PMAA/PNIPAM-2 microgels prepared with the fully swollen PMAA cores for thicker PNIPAM shells, via emulsion precipitation copolymerization in water, influenced higher drug-loading capability.	[190]
PNIPAM/HOOC-PNIPAM)-b-poly(2-(dimethylamino) ethyl acrylate)-C_12_H_25_ (HOOC-PNIPAM-b-PDMAEA-C_12_H_25_)/HOOC-poly(2-dimethylamino)ethyl acrylate)-b-PNIPAM)-C_12_H_25_ (HOOC-PDMAEA-b-PNIPAM-C_12_H_25_)	-	Sequential reversible addition-fragmentation chain transfer (RAFT) polymerization	The diblock copolymers were chemically modified to strong cationic, double hydrophilic, block polyelectrolytes via quaternization reaction on the PDMAEA block. The quaternized block copolymers form larger aggregates than the amine-based block copolymers because of the electrostatic repulsions of the positively charged quaternary amine groups.	[191]
PNIPAM/chitosan-poly(methacrylic acid) Cs-PMAA	-	Free radical emulsion polymerization	Copolymerized chitosan with MAA along with NIPAM is an improved version of chitosan gel to be further receptive to the atmosphere of the human body, including different pH, ionic strength, temperature, electric field, and enzyme activities. The small size of the particles is essential to ensure that the particles get through to the target site, especially in drug delivery.	[192]
PNIPAM/carboxymethyl chitosan/multiwalled carbon nanotube	Doxorubicin	In situ crosslinking polymerization	The hydrogels demonstrated dual-responsiveness of pH and temperature, and high maximal swelling ratios were possessed by multiwalled carbon nanotubes (MWCNTs)–COOH. The hydrogel could be utilized for the site-specific direct delivery of protein or hydrophilic anticancer drugs.	[193]
PNIPAM/3-(methacryloxypropyl)trimethoxysilane)	ibuprofen	Grafting and polymerization	The hybrid nanoparticles were monodispersed in an aqueous medium and displayed temperature dependency of standard hydrodynamic diameter, promoting them as drug nanocarriers. They demonstrated the exceptional temperature-regulated delivery of the model drug. Specifically, a low % release of ibuprofen below LCST along with a complete and fast ibuprofen delivery at higher than LCST contrasted the earlier report.	[177]
PNIPAM/poly(2-(dimethylamino) ethyl acrylate)20-b-PNIPAM)11-b-poly(oligo ethylene glycol methyl ether acrylate)18 (PDMAEA20-b-PNIPAM11-b-POEGA18)	-	Sequential reversible addition-fragmentation chain transfer polymerization	The thermoresponsive behavior was displayed by amine-based triblock terpolymer, despite the low amount of PNIPAM block in comparison to other comprising blocks. The chemically altered triblock terpolymers self-assemble into larger aggregates in the whole temperature scale compared to the amine-functionalized triblock terpolymer as a result of electrostatic repulsions of the permanently charged quaternary amine groups of the modified PDMAEA blocks.	[191]
PNIPAM/N,N-dimethylacrylamide (DMA)	-	Reversible addition-fragmentation chain transfer (RAFT) polymerization	The synthesis of six NIPAM and DMA-based statistical, ABA triblock, and ABABA pentablock copolymers for each comprised one or two dodecyl hydrocarbon end-groups. The results demonstrated extraordinary and carefully balanced tradeoffs among short non-polar end groups and customized hydrophobicity in the nanoscale self-fabrication of PNIPAm-based copolymers in the water near the LCST.	[194]
PNIPAM/poly(2-(4-formylbenzoyloxy) ethyl methacrylate)	Doxorubicin	Disulfide linkages	Shells of disulfide-bonded temperature-sensitive block copolymers act as gatekeepers to control drug release. The developed multifunctional materials do not produce premature release in blood circulation but accelerate drug release inside cancer cells.	[195]
PNIPAM/polyglutamic acid (γ-PGA)/polyethylene glycol (PEG)	-	Polymerization	The optimal mass ratio of comonomers (NIPAM, γ-PGA, and PEG), crosslinker, and initiator was secured at 1:0.2:1:0.01:0.01, defined by the response surface method (RSM). It was also discovered by RSM that the ESR was considerably reliant on the crosslinker along with the collaboration amongst the initiator and γ-PGA.	[196]
PNIPAM/polystyrene (PS)	-	Anionic polymerization	Thermoresponsive wetting performance as a role of substrate micromorphology with the surface. PS/PNIPAM films of various fusions were spin-casted on microstructured silicon substrates together with or devoid of a native SiO_2_ layer, and take up the benefit of the large specific area of the silicon substrates to enrich the film thermoresponsiveness.	[197]
PNIPAM/poly (stearyl methacrylate)	-	Reversible addition-fragmentation chain-transfer (RAFT) polymerization	The triblock copolymer micelles demonstrated a distinctive evolution, initially developing into small, then developing into larger, and finally stable. The transition process was fast as well as reversible with temperature. The hydrophobic PSMA chain segment dropped the LCST of the diblock copolymer micelles.	[198]

**Table 2:** Current investigations on PNIPAM-based hydrogels in gene delivery.

Device	Model Drug	Composition	Preparation Technique	Applications	Results	References
Hydrogels	RALA, plasmid DNA (p-DNA)	Alginate (Alg) grafted PNIPAM (Alg-g-PNIPAM)	Free radical polymerization	Castrate-resistant prostate cancer (CRPC)	The copolymer’s alginate backbone significantly influenced the mechanical and structural properties of hydrogels. At 37 °C, high-pitched MW alginate improved the copolymer’s rigidity, and the M/G ratio affected rigidity as well as the molecular network. In contrast to uncomplexed pDNA, which had a significant rupture release during the first six hours in Alg-g-PNIPAM hydrogels, RALA/pDNA NPs had a prolonged and controlled release over time. This offers up a slew of possibilities for remedial pDNA delivery from this thermoresponsive hydrogel, which proved to have a wide range of medical applications.	[212]
Thermoresponsive hydrogel	RALA/pEGFP–N1	Chitosan-g-PNIPAM crosslinked with genipin	Free radical polymerization	-	The proportion of chitosan in the copolymer affected the hydrogel’s breakdown, swelling, NP release level, and storage modulus. The Cs-g-injectability PNIPAM’s at room temperature suggested that it may be delivered to the target site in a minimally invasive manner. The hydrogel’s ability to provide long-acting drugs to target tissues was demonstrated by sustained NP release and breakdown over three weeks. More crucially, the nucleic acid payload remained active, as evidenced by the NCTC-929 fibroblast cell line’s excellent transfection.	[218]
Thermosensitive hydrogel	Irinotecan (CPT-11)/cetuximab (CET) conjugate graphene oxide (GO) (GO-CET/CPT11), stomatin like protein 2 (SLP2), and short heparin RNA (shRNA)	Chitosan-g-PNIPAM (CPN)	Free radical polymerization	Glioblastoma multiforme	Controlled drug release and increased mechanical strength of the in situ-produced hydrogel were achieved by combining a negatively charged nanocarrier and a positively charged CPN. CPT-11 release from a drug-loaded hydrogel exhibited a 28-day continuous release pattern, whereas the intricate shear modulus rose fivefold after entrapping GO-CET in the hydrogel. The formulation increased anti-tumor activity in vitro by eliciting a 53% apoptotic rate in 2 days. A xenograft tumor model was used to illustrate treatment efficacy, with a 40% reduction in tumor size after 12 days compared to the untreated control group.	[216]
Thermosensitive mesoporous silica nanoparticles (MSN)	microRNA-222 and aspirin (ASP)	Poly(ethylene glycol)-b-poly(lactic-co-glycolic acid)-b-PNIPAM (PEG-PLGA-PNIPAM)	Atom transfer radical polymerization and ring-opening copolymerization	Bone tissue engineering	As previously reported, ASP stimulated bone production, and miR222 triggered Wnt/-catenin/nemo-like kinase signaling to drive differentiation of bone mesenchymal stem cells to neural-like cells. Injection of co-delivered MSN hydrogel into a rat mandibular bone defect resulted in neurogenesis and faster bone development, suggesting that the injectable ASP and miR222co-delivering colloid hydrogel has potential for vascularized BTE.	[219]
Thermoresponsive hydrogels	SiRNA, glyceraldehyde-3-phosphate dehydrogenase (GAPDH)	PNIPAM/MgAl-layered double hydroxides (LDHs) (MgAl-LDH)	Radical polymerization	Degenerative disease of cartilaginous tissues	When the temperature of the hybrid hydrogel was increased from 25 to 37 degrees Celsius, it transitioned from a fluid to viscous gel phase in less than 10 s. The introduction of siRNA against a housekeeping gene into an in vitro model of cartilaginous tissue degeneration comprised of osteoarthritic cells was reported to achieve gene silencing in situ for 6 days with a high gene silencing efficacy (>80%). Providing extracellular matrix scaffolds and interfering with degenerative factor expression, therapeutic RNA oligonucleotides with supporting hydrogel material may offer promises in treating cartilaginous tissue degeneration.	[220]
Nanogels	Green fluorescence protein (GFP) gene, amine functional magnetic iron oxide nanoparticles (NH2-MNP)	PNIPAM- co-acrylic acid (p(NiPAAm-co-AAc)) coated with poly (ethyleneimine) (PEI)	Free radical polymerization	Gene delivery	Treatment with 20 mg/mL PEI-coated nanogels resulted in the maximum EGFP expression. After 24 h of transfection, EGFP expression was found for the first time, lasting up to 72 h. In hMSCs, self-assembled p(NiPAAm-co-dAAc) nanogels conjugated with the GFP gene were strongly expressed, suggesting they may be used for gene delivery.	[210]

**Table 3:** Recent investigations on PNIPAM-based hydrogels for tissue engineering.

Device Type	Model Drug	Polymer Formulation	Preparation Method	Applications	Results	References
Injectable hydrogel	Melatonin	PNIPAM/hyaluronic acid (HA) loaded chitosan-g-acrylic acid-coated PLGA (ACH/PLGA)	Single emulsion solvent evaporation	Cartilage tissue engineering	This system demonstrated excellent integration with genuine cartilage, and scanning electron microscopy pictures revealed an interconnected permeable structure. The hydrogels had exceptional MTT plus biocompatibility, and the live–dead assay demonstrated that WJMSCs could proliferate and survive. Overall, this injectable hydrogel proved to be an encouraging system for cartilage tissue engineering due to its increased mechanical properties, reduced syneresis, ability to sustain drug release, and high bioactivity.	[188]
Hydrogels	Mesenchymal stem cells (MSCs)	PNIPAM/chitosan	Freeze drying	Cartilage tissue engineering	The hydrogel solution’s residence duration inside the scaffold was determined to be 6 min for CSNI100 and 9 min for CSNI400. The swelling ratio of hybrid scaffolds was larger than that of chitosan-only scaffolds, and CSNI400 had a greater swelling ratio than CSNI100. In CSNI100 and CSNI400, the number of MSCs climbed by 58 and 108%. These findings imply that chitosan solid and PNIPAM hydrogels with a polymerization degree of 400 are found to be encouraging for cartilage tissue engineering.	[263]
Injectable hydrogel	Human dental pulp stem cells (hDPSCs)	PNIPAM-based copolymer/graphene oxide (GO)/chitosan (CS) crosslinked by beta glycerol phosphate (beta-GP) and genipin (GN)	Free radical copolymerization	Bone tissue engineering	Based on MTT, DAPI staining, and cell survival findings, the produced hydrogels provided a biomimetic ECM milieu for hDPSC proliferation and can be used as a novel BTE scaffold with good biocompatibility. The hydrogels ramped up the expression of osteogenic genes such as OCN and Runx 2, and activity of ALP and calcium deposition was enhanced.	[264]
Hydrogel	Oxacillin	PNIPAM/hydroxyapatite (HAp)	Electrochemical polymerization	Bone tissue engineering	The PNIPAM-HAp scaffolds were found to be very porous using SEM, and the HAp concentration appeared to govern the composite’s porosity. The scaffolds had original ingredients (no new chemical compounds were produced), and the ECP procedure did not affect the crystallinity of the HAp, according to XRD and FTIR analyses. Compared to the scaffolds with limited HAp content, the PNIPAM-HAp scaffolds with higher HAp content had a decreased oxacillin drug release rate. The oxacillin delivered from scaffolds maintained bacterial activity against P. aeruginosa and S. aureus for an extensive period. ECP seems to be a favorable methodology for producing PNIPAM-HAp scaffolds for BTE based on the data acquired from the above results.	[246]
Hydrogel	-	PNIPAM/cardiosphere derived cells (CDCs)	Free radical polymerization	Cardiac tissue engineering	Under static and dynamic stretching, the CDCs validated elastic modulus-dependent cardiac diversity, as revealed by gene and protein expressions of cardiac markers such as cTnI, Connexin43, CACNA1c, and MYH6. The expression of cardiac markers CACNA1c and MYH6 was considerably enhanced after 1 Hz frequency was applied to murine CDCs, indicating that they were driven to differentiate into cardiac lineage. In 40 kPa and 21 kPa hydrogels, the strain promoted CDC cardiac differentiation. These findings suggest that employing a 40 kPa hydrogel to transplant CDCs could result in optimum cardiac regeneration and differentiation.	[265]
Thermosensitive hydrogel	Brown adipose-derived stem cells (BASCs)	PNIPAM/single wall carbon nanotubes (SWCNTs)	Lyophilization	Cardiac tissue engineering	In vitro, SWCNTs with PNIPAM hydrogel demonstrated significantly more bioactivities to encapsulated cells than onefold PNIPAM. When utilized as a carrier, the technique improved seeded cell engraftment and survival in infarct myocardium, showing therapeutic efficacy following myocardial infarction.	[266]
Stimuli-responsive hydrogel	5-amino salicylic acid (5-ASA) and ornidazole	PNIPAM/glycogen (Gly) (cl-Gly/PNIPAM) and crosslinked by ethylene glycol dimethacrylate (EGDMA)	Free radical polymerization	Intestinal tissue engineering	The produced hydrogel’s LCST was reported to be in the spectrum of 32.5–34 °C. The hydrogel was shown to be compatible with human mesenchymal stem cells (hMSCs). The medications were efficiently loaded into the hydrogel system, which released both medications in a controlled manner, with 96–97% of the medications remaining stable after two months. The created hydrogel could be used for colon-focused delivery because of the nature and component stability of the medications.	[267]
Thermoresponsive hydrogel	-	Polyacrylic acid (PAA)/norbornene-functionalized chitosan (CsNb) crosslinked by bistetrazine-PNIPAM (bisTz-PNIPAM)	reversible addition–fragmentation chain transfer (RAFT) polymerization	Intestinal tissue engineering	The Tz-Nb click reaction between bisTz-PNIPAM crosslinker and CsNb created chemical crosslinks that improved the hydrogel structure’s durability and produced pores in the hydrogel grid that allowed high drug load capacity and release. Because of the pH responsiveness of PAA, shrinkage behavior, and hydrogel porosity of PNIPAM, the hydrogel only gave a restricted medication release (8.5%) of 5-ASA at pH 2.2, but then it showed practically perfect delivery (92%) at pH 7.4 and 37 °C within 48 h. The hydrogels were nontoxic to human fibroblast cells and were biodegradable, indicating that they have a lot of potential as a medication delivery mechanism for the colon.	[268]
Hybrid hydrogel	Chlorhexidine diacetate	P-methacrylate arginine (M-Arg)/*N*-isopropylacrylamide (NIPAAm)/polyhexamethylene guanidinie phosphate (PHMG) [P(M-Arg/NIPAAm/PHMG)] crosslinked by N-N’methylene bisacrylamide.	Free radical copolymerization.	Wound dressing	Changing the monomer’s mass ratio controlled the hydrogels’ mechanical characteristics, swelling manner, and CHX release in vitro. The zwitterionic M-Arg monomer validated the hydrogel device’s resilience to protein adsorption. The hydrogels’ wound healing performance and safety were validated in an in vivo and cytotoxicity investigation. Ultimately, this research showed that hydrogels that possess long-term, anti-protein adsorption and antibacterial capabilities could effectively heal wounds.	[269]
Thermosensitive hydrogel	Superoxide dismutase (SOD)	PNIPAM/poly (γ-glutamic acid)	Free radical polymerization.	Wound dressing	The hydrogels had thermo-sensitivity at physiological temperature, and the phase transition temperature was 28.2 °C according to results from a differential scanning calorimeter and gelling action. SOD activity in vitro reached up to 85% after 10 h, which seemed beneficial in eradicating the superoxide anion. MTT experiments ensured that this hydrogel was biocompatible. The thermo-sensitive hydrogels had a longer-lasting SOD release, improved moisture retention, and higher water absorption. The device has significant application potential for wound repair and may effectively stimulate healing.	[270]

## Data Availability

No new data were created or analyzed in this study. Data sharing is not applicable to this article.
